# An intricate relationship between fibrosis and autoimmune diseases: a specific focus on unraveling its molecular, immunological, and epigenetic drivers

**DOI:** 10.3389/fimmu.2026.1754620

**Published:** 2026-04-10

**Authors:** Manoviraj Gajendra Morey, Gouri H. Illanad, Mahaboobkhan Rasool

**Affiliations:** Immunopathology Lab, School of Biosciences and Technology, Vellore Institute of Technology (VIT), Vellore, Tamil Nādu, India

**Keywords:** autoimmunity, epigenetic regulation, extracellular matrix (ECM), fibrosis, TGF-β signaling

## Abstract

Fibrosis is a pathological process of wound healing, characterized by excessive deposition of extracellular matrix (ECM) and chronic activation of fibroblasts, leading to organ scarring and a decline in function. Fibrogenesis is predominantly initiated by tissue injury and sustained inflammation, further driven by the complex interplay of growth factors, cytokines, metabolic alterations, and epigenetic reprogramming. Activated myofibroblasts and immune cells function as primary profibrotic mediators. The central molecular pathways implicated include TGF-β/SMAD signaling, non-canonical cascades such as RAS-ERK and PI3K-AKT-mTOR, and integrin-mediated mechanotransduction. These pathways collectively contribute to matrix disruption by upregulating α-SMA, collagen, lysyl oxidase, and tissue inhibitors of metalloproteinases (TIMPs). Alterations in noncoding RNAs and histone/DNA modifications stabilize genes responsible for pro-fibrotic pathways, whereas metabolic reprogramming sustains myofibblast activity. These mechanisms contribute to fibrosis in several autoimmune disorders, including rheumatoid arthritis, multiple sclerosis, Sjögren’s disease, and Crohn’s disease. In these conditions, persistent immune activation drives continuous crosstalk between immune cells and stromal fibroblasts, sustaining cytokine signaling and ECM remodeling. Current therapeutic approaches primarily aim to halt disease progression; however, achieving true reversal remains challenging. The significant morbidity and mortality associated with fibrotic diseases underscore the need for clinically validated biomarkers to guide effective combined therapeutic regimens capable of reversing established scarring. In this review, we explored emerging strategies that emphasize the integration of immune modulation, epigenetic reprogramming, and mechanobiological interventions to inhibit myofibroblast proliferation and facilitate matrix degradation and tissue regeneration.

## 1 Introduction

Fibrosis is characterized by the dysregulation of the normal wound healing sequence, which is initially intended to maintain organ integrity. Therefore, a mechanism originally designed for healing transforms into one that drives pathological scarring (1). The association with autoimmunity is evidenced by chronic inflammation, leading to fibroblast activation and the accumulation of ECM components, particularly collagen and fibronectin, which disrupt the normal tissue architecture and function (2). The structural integrity of the tissue is compromised, resulting in pathological remodeling observed in rheumatoid arthritis, systemic lupus erythematosus, and crohn’s disease. Consequently, the regulatory balance shifts towards the persistence of the fibrotic response rather than its resolution. Therefore, the balance between collagen deposition and degradation is a critical aspect of wound healing (3, 4). Traditionally, chronic inflammation is regarded as the primary catalyst of fibrosis. However, recent studies have challenged this notion by emphasizing the symbiotic relationship between immune dysregulation and pro-fibrotic signaling, which perpetually exacerbate each other in a self-sustaining destructive cycle (2). Inflammatory mediators not only initiate fibrosis, but also actively perpetuate fibroblast activation and ECM production, thereby reinforcing inflammation in a feed-forward destructive loop. The role of initial stimuli, such as cytokines, chemokines, and autoantibodies, extends beyond merely initiating inflammation; it also sustains a persistently activated state of fibrogenesis (5, 6). Myofibroblasts, the primary effector cells for ECM deposition, are central to this pathological progression. They originate from multiple cellular sources, such as resident fibroblasts, epithelial/endothelial cells, and bone marrow-derived fibrocytes (7). Their differentiation is driven by elevated levels of specific growth factors, such as fibroblast-like growth factors (FGFs) and platelet-derived growth factors (PDGFs), which facilitate fibroblast–myofibroblast (FMT) transition. Key central pathways that facilitate this transition include transforming growth factor-β (TGF-β), WNT, MAPK, JAK/STAT, and Hedgehog (Hh) signaling (8).

The inflammatory stage is initiated by damage-associated molecular patterns (DAMPs) released by parenchymal cells at the site of injury. Coordinated interactions between immune cells and fibroblasts play a critical role in determining wound healing outcomes (1, 8). The disintegration of this meticulously regulated system is a characteristic feature of fibrosis, marked by the inability to resolve the inflammatory phase (5), which subsequently leads to the persistent activation of fibroblasts. These cells are distinguished by their ability to maintain an activated phenotype, achieving autonomy from external stimuli due to epigenetic modifications influenced by profibrotic factors, such as TGF-β (9). A pivotal stage is reached following the transition of epithelial cells into mesenchymal cells, driven by upregulation of FGFs and PDGFs (8). Activated myofibroblasts drive ECM deposition, transitioning from a reparative process to chronic fibrosis. Under normal physiological conditions, apoptosis, facilitated by the depletion of PDGFs and TGF-β, is responsible for the clearance of activated fibroblasts, thereby balancing tissue homogeneity (10, 11). However, the failure of this critical regulatory mechanism results in the persistent activation of these cells, thereby leading to progressive ECM deposition that further perpetuates cell damage (8).

This dysregulation is mechanistically reflected by a failure to resolve the tightly regulated stages of wound healing, which usually proceed through well-coordinated events of inflammation, proliferation, and remodeling (12). In rheumatoid arthritis, fibroblast-like synoviocytes (FLS) acquire an invasive phenotype that secrete matrix metalloproteinases (MMPs), facilitating ECM degradation and culminating in cartilage erosion. Single-cell transcriptomic analysis has identified the hyperactivity of FLS cells and the development of a proinflammatory phenotype, resulting in the recruitment of immune cells that perpetuate synovial inflammation (13). This situation is further aggravated by TGF-β signaling, which facilitates a fibrotic phenotype through the induction of type I, III, and VI collagens (14). In Sjögren’s disease, the salivary glands are primarily affected and serve as targets for autoimmune attacks that result in inflammation (15). The initiation and propagation of this process is facilitated by the release of IL-13 and IL-22. Conversely, myofibroblasts contribute to the release of TGF-β1, IL-1β, IL-6, vascular endothelial growth factor (VEGF), and IL-33, while exerting regulatory control over epithelial-mesenchymal transition (EMT) process (16). Fibrosis also constitutes a fundamental aspect of the diffused form of systemic sclerosis, also known as scleroderma (17). An increase in the synthesis and accumulation of collagen type I, III, V, and VII has been observed in the reticular dermis of the lesional skin of patients with systemic sclerosis (SSc) (18). Crohn’s disease is characterized by the formation of intestinal fibrosis, which is facilitated by the overexpression of ECM components secreted by myofibroblasts (19).

This review aims to elucidate the complex relationship between fibrosis and autoimmune diseases, with particular emphasis on identifying the molecular, immunological, and epigenetic mechanisms involved. Furthermore, the review will conclude by highlighting current and emerging therapeutic strategies and clinical trials designed to disrupt the cycle of inflammation and scarring.

## 2 Initiation of autoimmune fibrosis: tissue injury and immune sensing

Autoimmune-driven fibrosis is characterized by a failure in the resolution of wound healing, where ongoing immune-mediated injury triggers a transition to chronic inflammation. Initiation of autoimmune fibrosis unfolds through a coordinated sequence of stages: early damage sensing, innate immune amplification, and ultimately, adaptive immune polarization. This sequential recruitment of immune compartments converts acute tissue damage into a permanent state of ECM remodeling.

### 2.1 The initiation phase: early sensory recruitment

The initiation phase begins following injury with the release of DAMPs such as IL-1α, HMGB1, and ATP, in which IL-1α, an alarmin, activates fibroblasts and primes resident macrophages, thereby creating an initial inflammatory milieu prior to the establishment of adaptive immunity. Fibroblasts, the nearest responders, produce chemokines, including CCL2, CCL7, IL-6, and CCL8, which recruit monocytes and neutrophils into the tissue. Neutrophils are among the earliest arrivals and contribute to fibrosis through both proteolytic remodeling and immune crosstalk mechanisms. Upon activation, neutrophils undergo granule exocytosis, a process involving the secretion of cytoplasmic granules, particularly neutrophil elastase (NE) (20). A study investigating the impact of NE inhibitor sivelestat on bleomycin-induced pulmonary fibrosis revealed a significant reduction in fibrotic and inflammatory conditions within the lungs. Bleomycin generates free radicals that damage alveolar epithelial cells, resulting in inflammation and TGF-β-driven fibroblast activation, leading to collagen deposition and ultimately pulmonary fibrosis. These findings further imply inhibition of neutrophil elastase-mediated activation of TGF-β1, thereby demonstrating a functional connection between neutrophil elastase and the TGF-β signaling pathway (21). Fibrogenic conditions are exacerbated when protease inhibitors are inactivated, which subsequently influence the regulatory balance between MMPs and TIMPs (22). They release neutrophil extracellular traps, serine proteases (cathepsin G and neutrophil elastase), and ROS, which mediate ECM degradation, which activating latent TGF-β and cleavage of pro-MMPs into their active forms (23). Activated MMPs further degrade ECM components, altering the structural environment and liberating growth factors such as TGF-β, FGF, and VEGF. Simultaneously, activated fibroblasts release CXCL8 and G-CSF, establishing a positive feedback loop that prolongs neutrophil survival (24). Monocytes recruited into the tissue differentiate into macrophages under IL-1, TNF- α, and GM-CSF signaling. Macrophage-derived IL-6, IL-1β, and TGF-β further activate fibroblasts and enhance chemokine and MMP production. TGF-β signaling drives fibroblast-to-myofibroblast transition (FMT), initiating α-SMA expression and collagen synthesis. The initiation phase therefore establishes TGF-β bioavailability and stromal responsiveness.

### 2.2 The amplification phase: signal amplification loop and immune-stromal reprogramming

At the amplification stage, macrophage plasticity becomes the central determinant of disease progression. Macrophages display context-dependent polarization into pro-inflammatory (M1) and pro-fibrotic (M2) phenotypes. The M1 phenotype is activated in response to Th1 cytokines, such as IFN-γ and TNF-α, and facilitates the secretion of pro-inflammatory cytokines, including TNF-α, IL-6, and IL-12, thereby promoting apoptosis and necrosis. Conversely, the M2 phenotype is induced by Th2 cytokines, such as IL-4 and IL-13, which possess pro-fibrotic properties (25). A defining feature of the amplification phase is macrophage reprogramming through intracellular signaling axes. In idiopathic pulmonary fibrosis, the GSK3β-TIP60-H3K27ac signaling axis upregulates SGK1 (serum and glucocorticoid-induced kinase 1) in lung macrophages, promoting macrophage reprogramming from M1 fibrotic phenotype to M2. This shift leads to excess CCL9 recruitment of Th17 cells, enhancing IL-17-mediated immune dysregulation and TGF-β secretion that ultimately drives pulmonary fibrosis. Neutrophils further contribute to immune amplification through interactions with macrophages. Wang et al. explored the macrophage-neutrophil axis, that promotes macrophage-to-myofibroblast transition, primarily inducing renal fibrosis. In renal fibrosis, neutrophil-derived NETs act as immunomodulatory signals that program macrophages toward a pro-inflammatory phenotype. Macrophages, acting as intermediates connecting innate and adaptive immunity, amplify local inflammation by orchestrating CD8^+^ post-sensing NETs. Recruited CD8^+^ cells act as profibrotic drivers by releasing granzyme B (GZMB), inducing tubular epithelial cell apoptosis, EMT, and FMT, collectively exacerbating renal fibrosis (26). Thus, amplification is defined by reciprocal immune-stromal reprogramming and stabilization of TGF-β-driven remodeling.

### 2.3 The adaptive polarization phase

The progression towards irreversible fibrosis is a consequence of adaptive immune skewing rather than innate activation alone. While innate immune activation initiates early TGF-β bioavailability and stromal sensitivity, the dominance of specific T-helper cell programs dictate whether a tissue resolves or progresses toward chronic ECM accumulation. By differentially modulating fibroblast behavior and macrophage effector functions, these T cell–driven networks establish the immunological threshold that triggers irreversible tissue scarring. Type 1 inflammation is mediated by Th1 lymphocytes, cytotoxic T cells, and cytokines such as IFN-γ and TNF- α and is mainly involved in innate resistance against intracellular pathogens. In addition, type 2 inflammation, which is mediated by Th2 lymphocytes, ILC2s, and cytokines such as IL-4, IL-5, and IL-13, contributes largely to fibroblast activation, myofibroblast accumulation, and ECM accumulation, hence its relevance in pathological fibrosis (27). Research indicates that fibrogenesis is strongly associated with Th2 polarization. IL-4 is central to Th2 differentiation and directly enhances fibroblast proliferation and collagen synthesis. Additionally, the Th2 cytokine IL-13 plays a role in inducing lung fibrosis through its effect on fibroblasts (28). The significance of IL-13 has been underscored in the context of bleomycin-induced pulmonary fibrosis, where its absence markedly reduces fibrotic conditions. It is characterized by Th2-skewed IL-13 over Th1 cytokines, such as IFN-γ. IL-13 primarily impairs collagen homeostasis while simultaneously increasing TIMP1 levels and inhibiting IL-1-induced MMPs expression. Additionally, IL-13 activates TGF-β and promotes C10 (macrophage-recruiting chemokine), thereby initiating an autocrine inflammatory loop (29). IL-5 indirectly reinforces fibrosis by promoting eosinophil expansion and suppressing the antifibrotic cytokine IFN-γ. A study examining liver fibrosis induced by *Schistosoma mansoni* in IL-5 knockout mice demonstrated a significant reduction in fibrosis, leading to the conclusion that IL-5 is essential for fibrotic process, as it suppresses the anti-fibrotic cytokine IFN-γ and facilitates the development of a Th2 cell response. Additionally, IL-5 increases the IL-13/IFN-γ ratio in tissues, and its induction of eosinophils enhances the presence of M2 macrophages and fibroblasts (30).

In contrast, Th-1-derived IFN-γ exerted anti-fibrotic effects in a study utilizing bleomycin-induced fibrotic mice. IFN-γ reduced collagen expression in the lungs by suppressing TGF-β mRNA, thereby decreasing the production of TGF-β protein. IFN-γ primarily suppresses the Bleomycin-induced upregulation of TGF-β in macrophages and epithelial cells, thereby reducing macrophage-derived TGF-β signaling. In a fibrotic environment, TGF-β derived from macrophages and epithelial cells acts on fibroblasts to induce pro-collagen αI and αIII, whereas IFN-γ interrupts this profibrotic circuit by downregulating TGF-β mRNA abundance, thus, exerting its anti-fibrotic effect by reprogramming macrophage-epithelial-fibroblast crosstalk (31). The anti-fibrotic role of IFN-γ in pulmonary fibrosis is defined by its capacity to inhibit FMT and normalize the MMP/TIMP balance. By counteracting TGF-β1, IFN-γ reduces the expression of MMP-2, MMP-3, and MMP-9, thereby preventing the activation of latent TGF-β1 and β-catenin signaling pathways (32). This activity underscores the reciprocal antagonism between Th1 and Th2 cytokines, which serves as a decisive factor in determining whether tissue repair resolves or progresses to chronic fibrosis.

**Figure 1:**
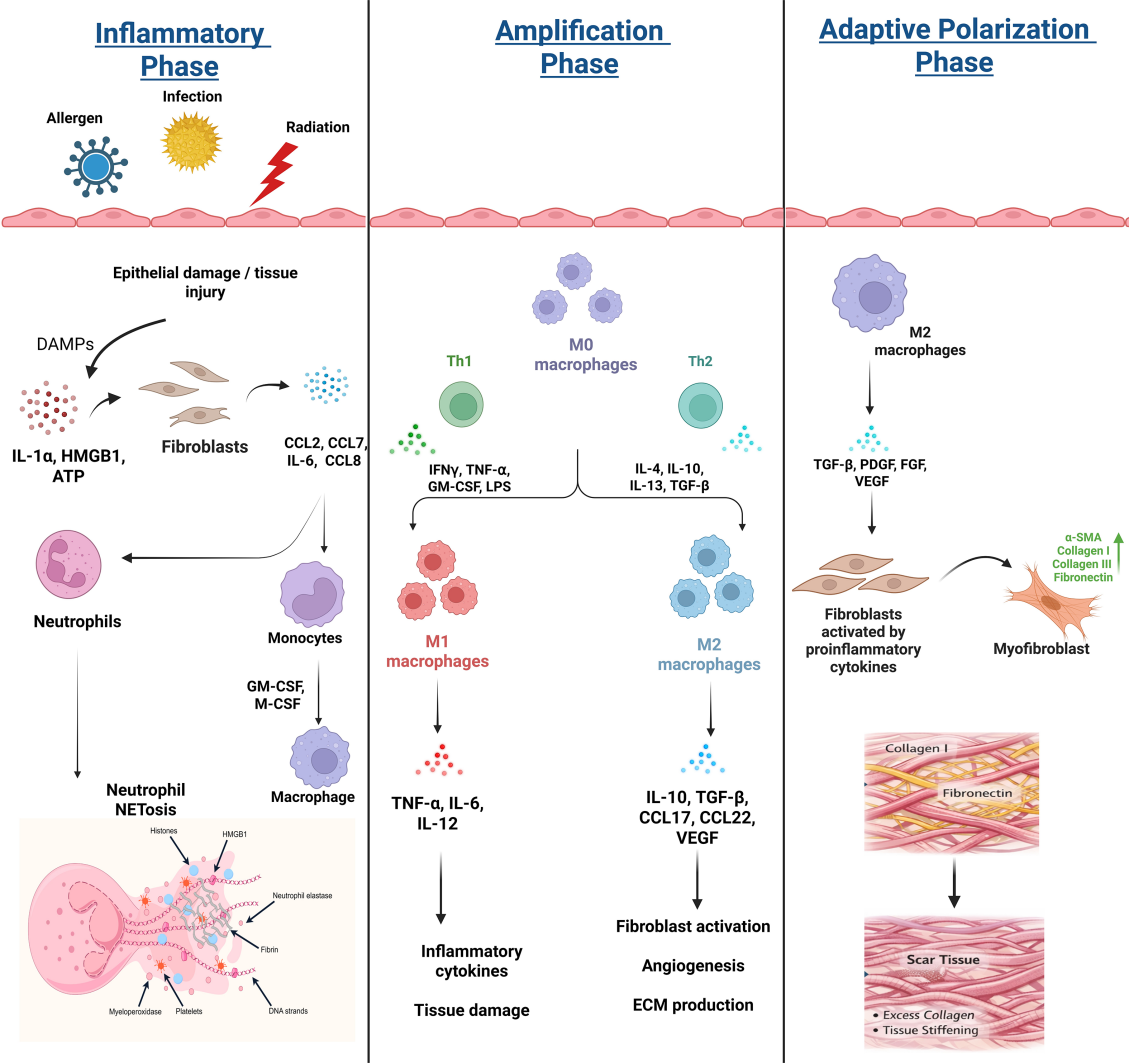
Schematic overview of the inflammatory, amplification, and adaptive polarization phases involved in fibrosis development. (Left) Initiation phase: Tissue damage caused by allergens, infection, or radiation triggers the release of DAMPs such as IL-1α, HMGB1, and ATP. They further activate fibroblasts, leading to chemokine (CCL2, CCL7, and CCL8) and IL-6 secretion, facilitating recruitment of neutrophils and monocytes. Activated neutrophils release NETs and neutrophil elastase to remodel the ECM and activation of latent TGF-β. Monocytes differentiate into macrophages that further enhance fibroblast activation and TGF-β signaling. (Middle) Amplification phase: Macrophages polarize into pro-inflammatory M1 or pro-fibrotic M2 phenotypes. M1 macrophages release TNF-α, IL-6, and IL-12, sustaining inflammation, whereas M2 macrophages produce IL-10, TGF-β, and VEGF, promoting fibroblast activation and ECM production. (Right) Adaptive polarization phase: Growth factors derived from M2 macrophages (TGF-β, PDGF, FGF, VEGF) stimulate FMT, wherein activated myofibroblasts express α-SMA. This results in an excessive deposition of collagen and fibronectin, which in turn causes scar formation and tissue fibrosis. DAMPs, Damage-associated molecular patterns; IL-1α, Interleukin-1 alpha; HMGB1, High mobility group box 1; ATP, Adenosine triphosphate; CCL2, C-C motif chemokine ligand 2; CCL7, C-C motif chemokine ligand 7; CCL8, C-C motif chemokine ligand 8; IL-6, Interleukin 6; NETs, Neutrophil extracellular traps; ECM, Extracellular matrix; TGF-β, Transforming growth factor beta; TNF-α, Tumor necrosis factor alpha; IL-12, Interleukin 12; IL--10, Interleukin 10; VEGF, Vascular endothelial growth factor; PDGF, Platelet-derived growth factor; FGF, Fibroblast growth factor; FMT, Fibroblast - myofibroblast transition; α-SMA, Alpha smooth muscle actin.

Regulatory T cells (Tregs), characterized by the presence of CD4+ CD25+ expression and Foxp3 transcriptional control, play a paradoxical role in fibrosis. While the primary function of Tregs is to maintain peripheral tolerance by suppressing excessive immune activation, their role in fibrotic pathology is highly context-dependent. Evidence from silica-induced injury model demonstrates that Tregs can actually promote fibrogenesis by suppressing IFN-γ production. Consequently, Treg depletion restores the anti-fibrotic Th1 program, highlighting a mechanism where the suppression of Th1-mediated resolution by Tregs allows for unregulated Th2-driven collagen synthesis. Treg cells exhibit a paradoxical antifibrotic function by modulating fibroblast recruitment to the injured area. The recruitment of fibroblasts and bone marrow-derived fibrocytes to the lung is mediated by the chemokine CXCL12, which operates through its receptor, CXCR4. A study conducted using a lung injury model with wild-type (WT) and lymphocyte-deficient Rag-1−/− mice demonstrated an increased fibrotic expression and collagen deposition compared to WT mice. Notably, when Rag-1−/− mice were administered with Tregs, there was a significant reduction in CXCL12 expression, ameliorating the fibrotic conditions (33). Another study reported an elevated accumulation of CD45+ ColIα1 fibrocytes following the depletion of Treg cells using an anti-CD25 antibody, resulting in a marked increase in cytokine levels and FGF-9. Consequently, it is postulated that Treg cells inhibit collagen deposition and fibroblast mobilization, at least partially, by suppressing FGF-9 (34) (**Figure 1****).** Collectively, adaptive immune polarization integrates cytokine signaling, transcriptional control, and immune–stromal reciprocity to determine fibrotic fate. The balance between Th1 resolution and Th2/Th17 progression is mediated by specific transcriptional anchors like STAT6 and STAT3. While Th1 cytokines counteract TGFβ to promote healing, sustained Th2 dominance triggers a self-reinforcing loop of collagen synthesis and macrophage activation. This “point of no return” converts transient repair into a permanent, self-perpetuating fibrotic state.

## 3 Cytokine amplification circuits driving fibrotic progression

**Figure 2:**
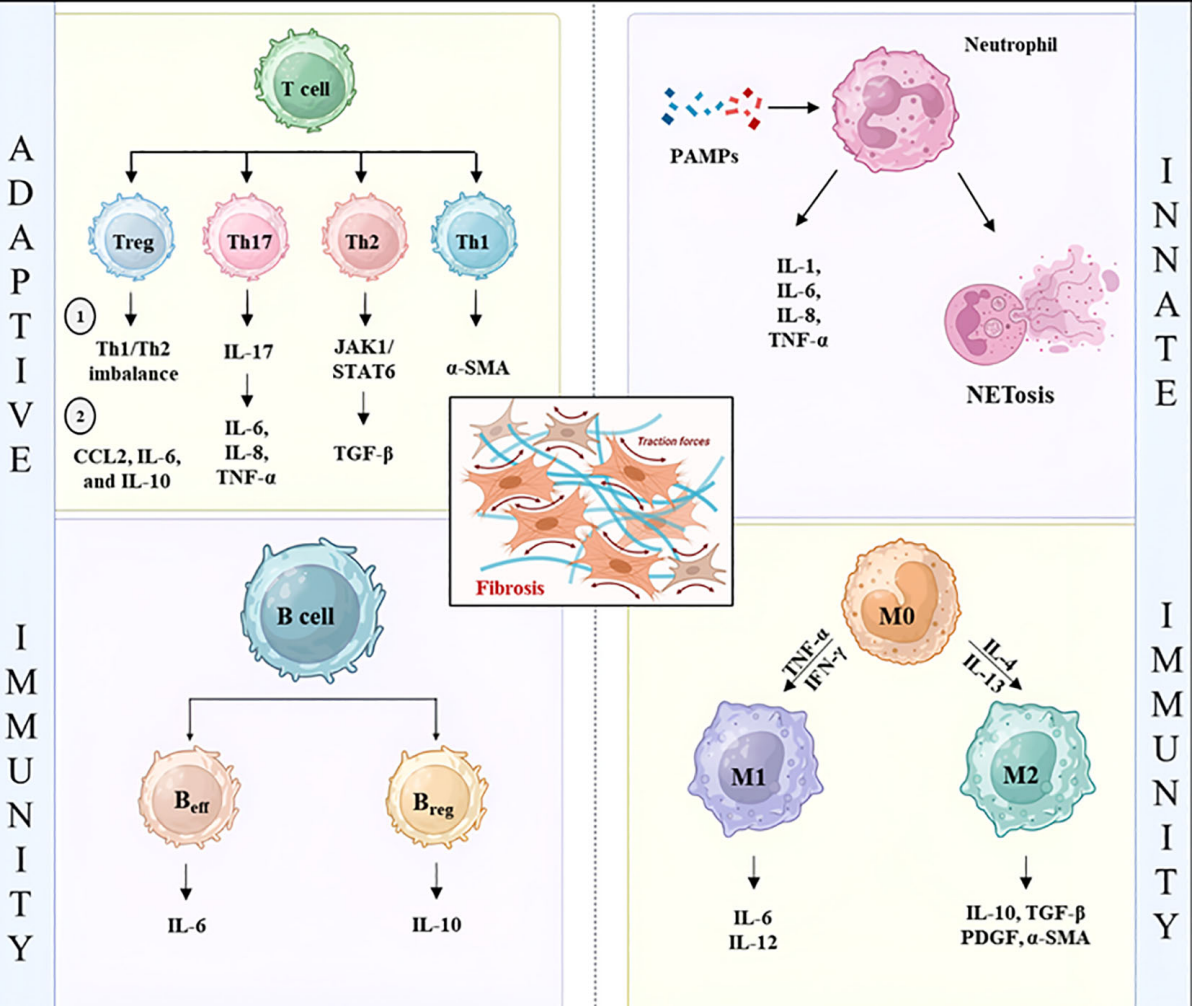
Immune cell interactions driving adaptive and innate responses that exacerbate fibrosis. (Left) Naïve CD4 cells differentiated into Th1, Th2, Th17, and Treg cell subsets in response to cytokines. Th1 cells produce IFN-γ and TNF-α, Th2 cells release IL-4, IL-5, and IL-13, Th17 cells secrete IL-17, and Tregs produce IL-10 and TGF-β. These cytokines play a key role in modulating B-cell activation and antibody production. (Right) Monocytes differentiate into M1 and M2 macrophages, primarily driven by IFN-γ, TNF-α, IL-4, and TGF-β. Neutrophils undergo NETosis, which amplifies the inflammation. IL-1β, Interleukin-1 beta; IL-4, Interleukin 4; IL-5, Interleukin 5; IL-6, Interleukin 6; IL-10, Interleukin 10; IL-13, Interleukin 13; IL-17, Interleukin 17; TGF-β, Transforming growth factor beta; IFN-γ, Interferon gamma; TNF-α, Tumor necrosis factor alpha; CCL2, C-C motif chemokine ligand 2; JAK1, Janus kinase 1; STAT6, Signal transducer and activator of transcription 6; α-SMA, alpha smooth muscle actin; ECM, Extracellular matrix; NETs, Neutrophil extracellular traps; FN, Fibronectin; p-SMAD, Phosphorylated SMAD proteins; Th1, T helper 1 cell; Th2, T helper 2 cell; Th17, T helper 17 cell; Treg, Regulatory T cell.

Autoimmune fibrosis begins with tissue damage and the recruitment of innate and adaptive immune cells. However, its progression is driven by self-perpetuating cytokine loops that lock inflammatory and structural cells into a permanent state of activation. This synchronization forces immune and stromal cells to continuously drive fibroblast activity, ensuring that the production of ECM never shuts off. Among these, several interconnected pathways have emerged as central drivers of fibrotic progression, including transforming growth factor-β (TGF-β) signaling, the IL-6/STAT3/IL-17 inflammatory axis, and type 2 immune pathways mediated by IL-4 and IL-13 (**Figure 2****).**

### 3.1 The TGF-β axis as the master profibrotic switch

TGF-β is a key signaling molecule comprising of three distinct isoforms: TGF-β1, TGF-β2, and TGF-β3, which serve as pivotal regulators of immune modulation, embryogenesis, and cellular proliferation. It functions as a critical mediator in the advancement of fibrosis, a pathological condition marked by the excessive formation of scar tissue (35–37). TGF-β belongs to a family of growth factors that includes bone morphogenetic proteins (BMPs), activins, growth differentiation factors (GDFs), and inhibins (38). In fibrotic settings, TGF-β plays a crucial role by interacting with TGF-β receptors, which are transmembrane proteins with intrinsic serine/threonine kinase activity (39). Following tissue injury or stress, necrotic death leads to the passive release of DAMP molecules, particularly IL-1α, into the extracellular space without the requirement for inflammasome activation (40). Acting as an alarmin, it binds to its receptor on neighboring cells like fibroblasts, triggering the release of MMP-2 and MMP-9, along with other matrix-remodeling enzymes, supporting its role as an upstream trigger of the proteolytic milieu required for latent TGF-β activation. MMPs are synthesized as inactive zymogens (pro-MMPs) and secreted into the extracellular space or localized to the cell surface, where they are proteolytically activated through coordinated protease cascades and interactions with membrane-associated molecules (CD44 and integrins) (41). Prior to activation and signaling, TGF-β exists in the ECM in its precursor form, bound to the latency-associated peptide (LAP), forming a complex known as latent TGF-β binding protein (LTBP) (42). Activation is facilitated by proteases such as MMP-2 and MMP-9, as well as thrombospondin, which cleave the LTBP complex, releasing the TGF-β homodimer to bind to its receptor and initiate signaling (43). Yu and Stamenkovic showed that MMP9 is recruited to the surface of the cell via its interaction with CD44. This leads to an increase in its protease activity, which results in the cleavage of LAP, thereby releasing the active form of TGF-β (44). Consequently, MMP2 cleaves the latent TGF-β complex after its recruitment to the cell surface by the αvβ3 integrin (45).

**Figure 3:**
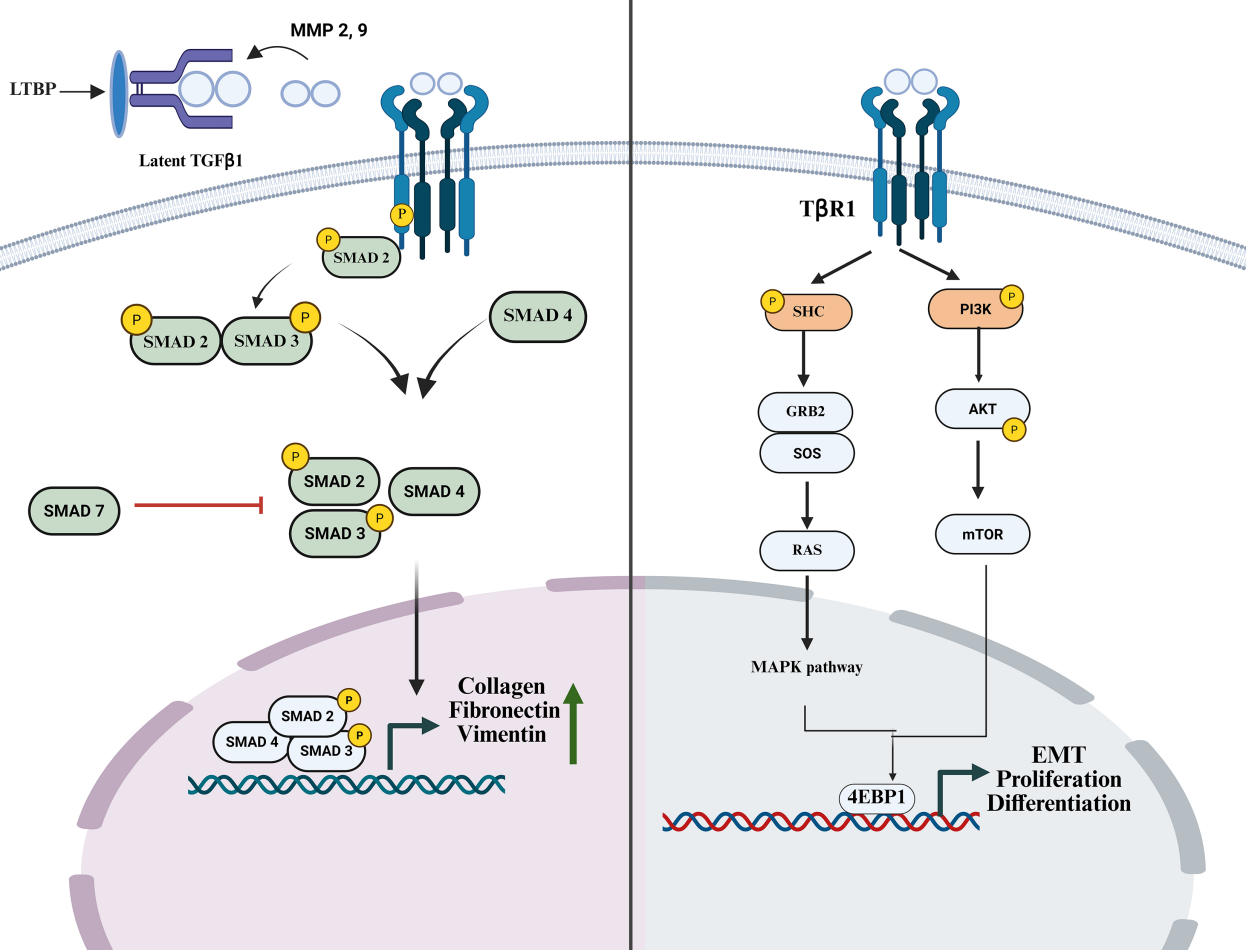
Schematic representation of canonical and non-canonical signaling pathways activated by transforming growth factor (TGF-β) and their roles in fibrosis. Upon ligand binding, TGF-β engages its receptor complex (TGF-βRI and TGF-βRII), triggering two major signaling pathways. (Left) Canonical SMAD-dependent pathway: Upon activation, phosphorylation of SMADs (SMAD2 and SMAD3) occurs. These form complexes with SMAD4, translocate to the nucleus, and upregulate pro-fibrotic genes, such as COL1A1, COL3A1, ACTA2, and ECM-modifying enzymes. (Right) Non-canonical (SMAD-independent) pathway: Transforming growth factor-beta (TGF-β) receptors also activate several parallel signaling cascades, including mitogen-activated protein kinase (MAPK) pathways (ERK, JNK, p38), PI3K/AKT, and NF-κB signaling. These activated pathways modulate fibroblast activation, migration, metabolism, and survival. Collectively, canonical and non-canonical TGF-β signaling integrate environmental cues to drive persistent inflammation, fibroblast activation, and progressive tissue fibrosis. TGF-β, Transforming Growth Factor-beta; SMAD, Mothers Against Decapentaplegic (homologs of Caenorhabditis elegans SMA genes and Drosophila MAD genes); PI3K, Phosphoinositide 3-Kinase; AKT, Protein Kinase B; GRB2, Growth factor receptor-bound protein 2; SOS, Son of Sevenless; RAS, Rat Sarcoma; 4EBP1, eIF4E-Binding Protein 1.

Upon activation, homodimeric TGF-β binds to TGFR2, which subsequently recruits and phosphorylates TGFR1 (also known as ALK5). The TGF-β signaling pathway consists of two primary pathways: canonical and noncanonical. In the canonical signaling pathway, activated TGFR1 phosphorylates Smad 2/3, which complexes with Smad4 and translocates to the nucleus to induce profibrotic gene transcription, leading to fibroblast activation, ECM deposition, and tissue scarring. This signaling is negatively regulated by inhibitory Smads, primarily Smad7, which acts as a regulatory brake on fibrosis by competing with Smad2/3 for receptor binding, thereby promoting receptor degradation (46, 47). In one such signaling pathway, TGFR1 phosphorylates SHC (Src homology 2 domain-containing transforming protein 1) at its tyrosine residues, facilitating the formation of a complex with GRB2 (growth factor receptor-bound protein 2) and SOS (son of sevenless homolog). This complex subsequently activates the RAS-ERK MAPK pathway, regulating cellular proliferation and differentiation (48, 49). The PI3K (Phosphoinositide 3-Kinase)-AKT pathway represents another significant non-canonical mechanism in the progression of fibrosis, particularly through its involvement in the EMT process, which can be inhibited to hinder fibrosis progression (50). This pathway facilitates mTOR activation via AKT phosphorylation, thereby promoting cellular responses such as growth, while concurrently inhibiting cell migration and invasion (51) (**Figure 3****).** TGF-β is a critical regulator in modulating the expression and activity of fibroblasts (52). It plays a pivotal role in EMT induction, marked by the downregulation of epithelial markers, E-cadherin and ZO-1, and the concurrent upregulation of mesenchymal markers, fibronectin and vimentin (53). Furthermore, TGF-β signaling induces the formation of a highly contractile fibrotic cell type by promoting the transition from fibroblasts to myofibroblasts (54). This process, known as the fibroblast-to-myofibroblast (FMT) transition, results in tissue scarring due to the upregulation of α-SMA protein in fibroblasts (55). The concentration of this protein is directly proportional to the contractile efficiency of individual fibroblasts (56). Persistent activation of the TGF-β signaling pathway is a molecular hallmark of organ fibrosis, particularly in diseases affecting the liver, lungs, and kidneys. Consequently, therapeutic strategies are directed towards inhibiting the components of this pathway, such as the TFGR1/ALK5 receptor, to alleviate tissue scarring.

### 3.2 The IL-6/STAT3/IL-17 feed-forward loop

A critical driver of fibrotic progression is the IL-6/STAT3/IL-17 signaling axis, a self-perpetuating loop that connects adaptive immunity to tissue remodeling. Central to this circuit is the differentiation and activation of T helper 17 (Th17) cells, which represent a distinct lineage characterized by the transcription factor RORγt and the secretion of cytokines such as IL-17A and IL-17F. The differentiation of this subset is primarily driven by the cytokines TGF-β and IL-6. Acting upstream of IL-23, IL-6 sustains the expression of the master transcription factor RORγt and promotes IL-23 receptor expression, thereby enabling Th17 cell maintenance. IL-6 also induces IL-21, which enhances IL-23 production and further consolidates lineage commitment. In the absence of IL-6, Th17 differentiation is significantly impaired, underscoring the importance of STAT3 signaling in fibrotic contexts (57). Th17 cells are integral to fibrogenesis and have been extensively investigated in various organs. In the context of liver fibrosis, IL-17 activates Kupffer cells, prompting them to secrete inflammatory cytokines, including IL-6, IL-1β, TNF-α, and TGF-β. Additionally, IL-17 influences hematopoietic stem cells to produce type 1 collagen by transforming them into myofibroblasts through the STAT3 signaling pathway (58). In lung fibrosis, exposure of CD4+ T cells to *S. rectivirgula* induces the differentiation of Th17 cells, characterized by the expression of IL-17 and IL-22. Notably, lung fibrosis is mitigated when IL-17 signaling is inhibited, underscoring its involvement in lung fibrosis (59). Previous research has demonstrated that TGF-β not only facilitates the differentiation of Th17 cells but also inhibits the differentiation of Th1 and Th2 cells (60). The reduced sensitivity of Th17 cells to the suppressive effects of T regulatory cells underscores their significant role in sustaining inflammatory responses (61). In pulmonary fibrosis, neutrophils serve as an early source of B cell-activating factor (BAFF), whose expression is induced by IL-1β and IL-17A signaling. Central to the neutrophil-Th17-BAFF axis is a reciprocal IL-1β-BAFF feedback loop that amplifies inflammation. IL-17A, upstream of BAFF, potentiates Th17 responses by amplifying IL-17A production in pulmonary T cells. Although not directly profibrotic, BAFF sustains a cytokine network that leads to TGF-β production, macrophage-dominated inflammation, and collagen deposition (62). Simultaneously, activated fibroblasts reinforce neutrophil persistence by releasing CXCL8 and G-CSF, prolonging neutrophil survival and sustaining recruitment.

B cells also contribute to the amplification of fibrotic signaling within this pathway. Beyond their role in adaptive immunity, they activate fibroblasts via PDGF-β and by secreting collagenous proteins (63). In pulmonary tissues, B cells are actively involved in the secretion of MMP-7, IL-17, IL-6, and VEGF, amplifying inflammatory and remodeling pathways. In patients with systemic sclerosis, B cell–fibroblast interactions induce IL-6, CCL2, and TGF-β1 secretion, driving collagen synthesis at levels comparable to recombinant TGF-β1 stimulation. Similar to T cells, B cells play a dual role in fibrosis progression through B effector cells (B_eff_) and B regulatory cells (B_reg_). A study examining these subsets in patients with SS identified significant dysregulation, wherein the frequency of B_eff_ cells (producers of IL−6) was elevated in the blood of patients compared to healthy controls. Concurrently, the frequency of B_reg_ cells (primarily producers of anti-inflammatory IL−10) was notably higher in patients. Consequently, the B_eff_/B_reg_ cell ratio was increased in patients with systemic sclerosis, which exhibited a positive correlation with the skin score, a clinical measure of fibrosis severity (64).

Together, these interconnected pathways establish a feed-forward inflammatory circuit where IL-6-driven STAT3 signaling promotes Th17 differentiation. Subsequently, IL-17 amplifies both immune and stromal activation, while auxiliary mediators such as BAFF and B- cell derived cytokines further reinforce the inflammatory milieu. This cumulative signaling environment bypasses regulatory checks to drive sustained myofibroblast differentiation and pathological ECM accumulation.

### 3.3 Type 2 immunity (IL-4/IL-13) and alternative macrophage programming

Beyond the Th17 inflammatory network, the fibrotic trajectory is heavily influenced by Type 2 immunity. Cytokines such as IL-4 and IL-13 are central to this process, directly promoting fibroblast activation, macrophage reprogramming, and progressive ECM accumulation. These mediators are primarily secreted by Th2 lymphocytes, Type 2 innate lymphoid cells (ILC2) and specialized immune populations during chronic inflammatory states.

Mechanistically, IL-4 and IL-13 engage with heterodimeric receptors composed of IL-4Rα, the common gamma chain (γc), and IL-13Rα1 subunits. The interaction of these cytokines with their respective receptors initiates the activation of Janus kinases (JAKs), specifically JAK1/JAK3 for type I receptors and JAK1/TYK/JAK2 for type II receptors. This activation triggers signaling cascades through the phosphorylation of tyrosine residues, ultimately leading to the activation of transcription factors such as Signal Transducer and Activator of Transcription 6 (STAT6) (65). The translocation of STAT6 to the nucleus facilitates the transcription of pro-fibrotic genes, including type I collagen and TGF-β, which promote fibrosis by driving ECM formation and tissue remodeling (66).

A defining feature of type 2 immunity in fibrotic disease is its ability to reprogram macrophages towards an alternatively activated M2 phenotype. Stimulated by IL-4 and IL-13 through a STAT6-dependent mechanism, these macrophages display characteristic markers including Arg-1, Ym1, and CD206 (67, 68). M2 macrophages produce a range of profibrotic mediators, including TGF-β, PDGF, and fibronectin, which collectively stimulate fibroblast activation and collagen synthesis. The interaction between alternatively activated macrophages and fibroblasts further reinforces fibrotic progression. Macrophage-derived TGF-β acts as a central mediator of FMT, promoting α-smooth muscle actin (α-SMA) expression and increased collagen deposition. In parallel, macrophage-derived growth factors such as PDGFs and connective tissue growth factor (CTGFs) enhance fibroblast proliferation and ECM production (69). These interactions create a microenvironment in which immune signaling and stromal activation mutually reinforce each other. Collectively, type 2 immune signaling represents a key cytokine circuit that drives fibrotic remodeling through IL-4- and IL-13-mediated STAT6 activation, macrophage polarization toward an M2 phenotype, and sustained fibroblast activation, this pathway contributes to the persistent deposition of ECM that characterizes chronic fibrotic disease.

Taken together, these distinct cytokines converge to establish an integrated immune–stromal signaling network that drives the persistence of autoimmune fibrosis. TGF-β activation serves as the primary molecular trigger that directly mediates fibroblast transformation and the synthesis of ECM. Parallel inflammatory circuits, particularly the IL-6/STAT3/IL-17axis, sustain immune activation and amplify cytokine production, while Type 2 immune responses mediated by IL-4 and IL-13 promote macrophage polarization toward profibrotic M2 phenotypes. By facilitating constant reciprocal crosstalk between the immune and stromal sectors, these pathways create a self-perpetuating feedback loop where inflammation, macrophage reprogramming, and myofibroblast activity are mutually reinforced.

## 4 Epigenetic modifications and genetic factors influencing fibrosis

### 4.1 DNA methylation

DNA methylation occurs at the fifth carbon of the CpG dinucleotide in the DNA sequence, catalyzed by an enzyme known as DNA methyltransferase (DNMT), resulting in the formation of 5-methyl cytosine (5MeC) (70). Two principal mechanisms have been proposed to elucidate the changes in gene expression due to DNA methylation: 1) The methylation process interferes with the interaction between transcription factors and their binding sites on the DNA. Some of these transcription factors include AP-2, c-Myc/Myn, and cyclic AMP-dependent activators CREB, E2F, and NFkB. 2) This mechanism involves the binding of specific transcriptional repressor molecules (MeCP1 and MeCP2) to methylated DNA at 5’-methylated CpG residues (71).

The MeCP2 gene is recognized for the normal development of the nervous system, with mutations in this gene associated with the onset of Rett syndrome and other X-linked disorders (72). Notably, its function extends to promoting myofibroblast differentiation in hepatic cells during fibrosis. This role was elucidated in a mouse model deficient in this protein, which resulted in reduced fibrosis, leading to the attenuation of liver injury. PPARγ, a transcription factor involved in lipid metabolism, glucose homeostasis, and inflammation, functions as a key suppressor of myofibroblast differentiation, and its repression facilitates myofibroblast activation. Hence, mice lacking the MeCP2 gene retain PPARγ expression, leading to reduced type 1 collagen production and fibrotic progression (73). This finding was corroborated by a study on liver fibrosis, where protein analysis indicated elevated expression of MeCP2 and α-SMA in diseased liver tissue (74).

DNA methylation is intricately associated with histone modifications, and both processes occur concomitantly. This interrelationship is hypothesized to be partially mediated by MeCPs (75). Another epigenetically regulated gene implicated in fibrogenesis through its role as a Ras inhibitor is RASAL1. Silencing RASAL1 expression promotes fibroblast activation and fibrosis. During repair, RASAL1 methylation is reversible, allowing for transient fibroblast activation. However, persistent fibrosis is driven by DNMT1-mediated hypermethylation of the RASAL1 promoter. This stable silencing is maintained by pro-fibrotic cytokines, including TGF-β1 (76). Additionally, a connection between MeCP2 and RASAL1 has been demonstrated in rats with hepatic fibrosis, wherein silencing of the MeCP2 gene in HSC-T6 cells inhibited fibroblast activation by upregulating the expression of RASAL1 (74). The role of MeCP2 has also been observed in cardiac fibrosis, promoting cardiac fibroblast proliferation via downregulation of DUSP5 (suppressing ERK1/2 phosphorylation, which promotes cellular proliferation). Silencing of this gene in neonatal rat cardiac fibroblasts leads to the inhibition of collagen I and α-SMA gene expression via upregulation of DUSP5 (77). Hypermethylation of RASAL1 (an inhibitor of Ras-GTP activity) is observed in the end stages of cardiac fibrosis, possibly due to its hypermethylation. However, a reversal of this aberrant methylation is observed via TET3-mediated hydroxymethylation induced by BMP7. This leads to the reversal of EndMT and a reduction in cardiac fibrosis (78).

### 4.2 Histone modifications

Chromatin constitutes the structural framework within which DNA is organized in the cell, involving the utilization of histone proteins around which DNA is coiled (79). These core histone proteins are the primary targets for modifications that regulate gene expression, such as methylation, acetylation, phosphorylation, ubiquitylation, SUMOylation, and ADP-ribosylation (80).

Hyperacetylation of histone proteins is correlated with enhanced gene expression, attributable to the increased accessibility of transcription factors due to DNA relaxation. This process is facilitated by a class of enzymes known as histone acetyltransferases (HATs) (81). The influence of HDACs was demonstrated by treating mouse hepatic stellate cells with valproic acid (an HDAC inhibitor), which suppressed their activation. This led to reduced LOX expression through the knockdown of class I HDACs, leading to a decrease in hepatic stellate activation. This attenuates fibrotic progression due to reduced ECM accumulation (82). HDACs have also been shown to regulate inflammatory gene expression by repressing MMP13. NF-κB regulates transcription by recruiting co-repressors, such as HDAC1. The p50 subunit forms anti-inflammatory homodimers that suppress MMP13 expression by recruitment of HDAC1, suppressing its gene expression by binding to the MMP13 promoter (83).

Myofibroblastic stellate cells demonstrate reduced acetylation of histones H3 and H4, along with increased expression of class II HDACs, particularly HDAC 4, relative to HDAC5 and 7. HDACs negatively regulate the expression of MMP genes, particularly MMP9 and 13. This was observed in the case of HDAC4, even in the presence of IL-1, which is known to markedly enhance the promoter expression of both MMPs (84). Epigenetic suppression of type I collagen in mouse liver hepatocytes undergoing TGFβ1-induced EMT was observed following the administration of the HAT inhibitor Trichostatin A interfered with the binding of p300 (HAT) to Smad3. This resulted in a reduction of TGFβ1-induced EMT (85).

Epigenetic regulators sustain fibrosis by converting transient TGF-β signals into permanently activated myofibroblasts. At the core of this transition, DNA methylation through DNA methyltransferase DNMT1 and MeCP2 has been shown to silence the anti-fibrotic genes PPARγ and RASAL1. More precisely, DNMT1 ensures the persistent silencing of RASAL1, which locks the fibroblast in an activated state, and Class I and II histone deacetylases alter chromatin accessibility to further promote continuous collagen expression.

### 4.3 Non-coding RNA activity

The coding regions of the human genome constitute only 2%, whereas the non-coding regions encompass the remaining portion (86). These include short non-coding RNAs, such as microRNAs (miRNAs), short interfering RNAs (siRNAs), piwi-interacting RNAs, and long non-coding RNAs (lncRNAs).

MicroRNAs play a crucial role in RNA silencing mechanisms by primarily targeting mRNA transcripts generated through transcription. Their function is facilitated by binding to the 3’ untranslated region (UTR) of mRNA transcripts, resulting in downregulation (87). In fibrosis, dysregulated miRNAs promote disease progression by acting as profibrotic and anti-fibrotic regulators. They facilitate this by targeting canonical and non-canonical TGF-β pathways, connective tissue growth factor (CTGF), ECM remodeling proteins, and EMT-related factors. CTGF and TGF-β reinforce each other’s gene expression (88). The miR-17–92 cluster comprises six miRNAs, among which miR-18a, miR-19a, and miR-19b have been identified as regulators of CTGF in aged cardiac cells. The upregulation of these miRNAs in cardiomyocytes leads to reduced CTGF expression. Conversely, low levels of these miRNAs are associated with increased CTGF expression (89). In cardiac fibroblasts and cardiomyocytes, miR-30 acts as a negative regulator of CTGF. By directly acting on the 3’-untranslated region (UTR) of the CTGF mRNA, miR-30 exerts a strong inhibitory effect. However, by downregulating miR-30, the expression of CTGF increases by more than 100% at the mRNA level and 300% at the protein level (90). miR-29 has been identified as a key regulator of ECM protein synthesis, significantly influencing the expression levels of type I and type III collagen. PDGF-B and TGF-β downregulate miR-29a, resulting in its upregulation and the establishment of a positive feedback loop that exacerbates profibrotic conditions (88). Similarly, miR-21 is instrumental in the pathogenesis of fibrosis in cardiac tissues. This is evidenced by the aberrant expression of miR-21 in cardiac fibroblasts, which is associated with a decrease in SPRY1 (an inhibitor of the Ras/MEK/ERK pathway) protein expression, leading to enhanced ERK-MAP kinase pathway activity. Consequently, this results in the persistence of cardiac fibroblasts, thereby exacerbating the fibrotic conditions during heart failure (91).

Thus, miRNAs have been demonstrated to significantly impact fibrosis and may serve as potential therapeutic targets. However, it is crucial to characterize the role of specific miRNAs (fibrotic or anti-fibrotic) when considering them as therapeutic targets. It is also essential to ensure that these miRNAs specifically affect only the intended target genes. The following epigenetic drivers have been identified for their respective mechanisms of action and their pro/anti fibrotic roles (**Table 1**).

**Table 1:** Epigenetic modifications driving organ-specific fibrosis.

Epigenetic modifying agent	Function	Fibrosis type	Fibrotic effect	References
Dna Methylation				
DNMT 1	Hypermethylates PTEN	Liver Fibrosis	Pro fibrotic	(92)
	Silencing of SOCS3	Cardiac Fibrosis	Pro fibrotic	(93)
DNMT 3A	Silences RASSF1A, Upregulation of ERK1/2	Cardiac Fibrosis	Pro fibrotic	(94)
	Klotho promoter hypermethylation	Renal Fibrosis	Pro fibrotic	(95)
DNMT 3B	Methylation of RCAN1.4	Liver Fibrosis	Pro fibrotic	(96)
	Activates Wnt/β-catenin pathway via suppression of SFRP5 expression	Renal Fibrosis	Pro fibrotic	(97)
Histone Deacetylases				
HDAC 1	Promotes TGF- β and EGFR signalling	Renal Fibrosis	Pro fibrotic	(98)
HDAC 2	HSC activation via suppression of SMAD7	Renal Fibrosis	Pro fibrotic	(99)
HDAC 3	Promotes EMT via Notch 1 and STAT1 signalling	Pulmonary Fibrosis	Pro fibrotic	(100)
Micro RNA				
miR-17-5p	Promotes HSC activation and proliferation by targeting SMAD7	Liver Fibrosis	Pro fibrotic	(101)
miR-21	Promotes ERK-MAP kinase pathway via reduction in SPRY	Cardiac Fibrosis	Pro fibrotic	(91)
miR-17-92	Downregulates CTGF	Cardiac fibrosis	Anti fibrotic	(89)
miR-141	Downregulates TGF-β and EMT	Kidney Fibrosis	Anti-fibrotic	(102)
miR-132	Decreases Myofibroblasts differentiation	Liver Fibrosis	Anti- fibrotic	(73)
miR-150	Downregulates the activation and proliferation of HSC	Liver Fibrosis	Anti- fibrotic	(103)
miR-192	Increases collagen synthesis	Kidney Fibrosis	Pro-fibrotic	(104)
miR-382	Upregulation in EMT	Kidney Fibrosis	Pro-fibrotic	(105)
miR-23a (clustered with miR-27a)	Upregulation in EMT	Pulmonary Fibrosis	Pro-fibrotic	(106)
miR-215	Upregulation in EMT	Kidney fibrosis	Pro-fibrotic	(107)
miR-200 family (miR-200a/b)	Downregulates TGF-β -dependent EMT	Liver Fibrosis	Anti-fibrotic	(108)
miR-155	Inhibits EMT and Erk pathway	Liver Fibrosis	Anti-fibrotic	(109)
miR-199a	Promotes TGF-β pathway and Fibroblast activation	Pulmonary Fibrosis	Pro-fibrotic	(110)
miR-129	Supresses STAT1 expression	Pulmonary Fibrosis	Pro-fibrotic	(111)
miR-133a	Decreases Collagen synthesis and ECM accumulation	Liver Fibrosis	Anti-fibrotic	(112)

The progression of chronic inflammation to fibrosis is driven by a coordinated network epigenetic cascade that creates a transcriptional memory. Inflammatory mediators such as TGF-β and NF-κB trigger DNA methylation, wherein DNMTs and MeCP2 silence critical anti-fibrotic genes such as PPARγ and RASAL1 and unlock the Ras/ERK signaling pathway. Concurrently, histone deacetylation by Class I and Class II HDACs remodels the chromatin, suppressing matrix-degrading enzymes while simultaneously facilitating collagen and α-SMA expression, driving myofibroblast differentiation. Finally, miRNAs integrate these signals, with profibrotic miR-21 enhancing fibroblast survival, while protective miRNAs, such as miR-29 and miR-30, are suppressed. This coordinated epigenetic locking prevents the restoration of homeostasis, driving the progression of chronic fibrosis across organ systems.

In addition to epithelial stress, epigenetic reprogramming sustains fibroblast activation. In fibrotic kidneys, induced TGF-β1 induces DNMT1-mediated hypermethylation and silencing of the RASAL1 promoter, a Ras-GTPase activator, resulting in persistent Ras pathway activation, which drives fibroblast proliferation, α-SMA expression, and collagen production. Demethylation or partial DNMT1 deficiency attenuates fibroblast activation via reduction of RASAl1 methylation (76). Thus, epigenetic locking of fibroblasts ensures the long-term maintenance of the fibrotic state, even after the initial insult has subsided.

## 5 Fibrosis and autoimmunity: a close relationship

Fibrosis represents a pathological endpoint of chronic inflammation in many autoimmune diseases. Persistent immune activation in autoimmune diseases drives chronic tissue inflammation, which ultimately culminates in fibrotic remodeling of affected organs. Cytokines, such as TGF-β, IL-6, and IL-17, play central roles in driving these processes by activating stromal cells and sustaining profibrotic signaling pathways.

### 5.1 Multiple sclerosis

Multiple sclerosis (MS) is a chronic autoimmune disorder characterized by immune-mediated demyelination, neuronal cell death, and axonal degeneration (113). Chronic CNS inflammation drives tissue remodeling via excessive ECM deposition, leading to neurofibrosis and glial scarring (114). Fibrosis, characterized by excessive ECM deposition, inhibits oligodendrocyte progenitor cell (OPCs) differentiation and remyelination, whereas fibronectin aggregates disrupt integrin-mediated signaling essential for maturation (115). Neurofibrosis is driven by the recruitment and activation of fibroblasts, microglia/macrophages and reactive astrocytes. IL-17 and IL-21 promote profibrotic progression, whereas IL-10 exerts protective and anti-fibrotic effects. IL-4, IL-6, and IL-13 play context-dependent roles in promoting pathological inflammation and neuroprotection. Astrocytes drive glial scarring by producing ECM components that inhibit axonal growth and remyelination. Their activation is linked to IL-17 signaling, where IL-17/IL-17R engagement via Act-1 recruits and ubiquitinates E3 ubiquitin ligase TRAF6, triggering IKK-dependent nuclear factor-kappa B (NF-κB) activation. This induces pro-inflammatory and pro-fibrotic factors, including IL-6, IL-8, CCL20, and MIP-1α (Macrophage Inflammatory Protein-1α) (116). MIP-1α recruits macrophages that release TGF-β, IL-1β, and PDGF, thereby activating fibroblasts and promoting collagen synthesis. CCL20-CCL6 signaling recruits Th17 cells that secrete IL-17 and TNF-α, sustaining a profibrotic feedback loop. These reactive astrocytes and perivascular fibroblast-like cells overproduce ECM proteins, including collagen and fibronectin, resulting in fibrotic glial scars (117). IL-17 upregulates glial fibrillary acidic protein (GFAP), marking astrogliosis in primary mouse astrocytes, an effect attenuated by MIP-1α neutralization. IL-1β upregulates P2X7 receptors in reactive astrocytes, resulting in Ca²^+^ influx and membrane pore formation. Excess Ca^²+^ levels activate enzymes and induce ROS-mediated mitochondrial dysfunction, leading to apoptosis (118, 119). Mason et al. demonstrated that IL-1β is critical for central nervous system (CNS) repair. In the cuprizone demyelination model, IL-1β-/- mice exhibited only 45.1 ± 1.6% axonal recovery, versus 67.1 ± 2.5% in wild-type mice. IL-1β expression increased during demyelination (weeks 3–6) and remained elevated during early remyelination (week 6), coinciding with the accumulation of IGF-1–producing cells and NG2^+^ OPCs. Thus, non-selective IL-1β suppression may impair repair, emphasizing the need for temporally controlled cytokine therapy (120). IL-10, which is regulated by Tregs and M2 macrophages, is a critical modulator of fibrosis. Although it promotes inflammation resolution and tissue repair via M2c macrophage polarization and controlled ECM formation, its sustained production drives chronic fibrosis via fibroblast recruitment and TGF-β-mediated collagen deposition, as observed in neurofibrotic MS lesions. This transition shifts acute inflammation toward persistent structural remodeling (121). Therefore, effective anti-fibrotic strategies require coordinated modulation of IL-10 and TGF-β signaling, as excessive TGF-β suppresses IL-10 and promotes ECM accumulation. Multiple sclerosis is therefore the result of a cascade of dysregulated autoimmune responses that promote fibrotic remodeling within the central nervous system. Effective therapeutic strategies therefore need to consider the modulation of autoimmune and fibrotic pathways rather than broad immunosuppression.

### 5.2 Rheumatoid arthritis

Rheumatoid arthritis (RA) is a chronic autoimmune inflammatory disorder characterized by joint inflammation, tissue proliferation, and progressive joint destruction, largely driven by IL-1, IL-6, and IL-17. Fibroblast-like synoviocytes (FLS) constitute the central component of the pannus, undergoing a transition from a quiescent state to an activated that facilitates irreversible damage through fibrosis. Notably, the FAPα+ THY+ FLS subset is associated with severe inflammation (122). One of the primary cytokines, IL-6 is temporally dependent; although it is crucial for early wound healing, chronic exposure may lead to pathological fibrosis. TGF-β1 induces IL-6 overexpression in the epithelial cells, facilitating EMT and FMT via a paracrine mechanism, whereas an increase in IL-6 enhances TGF-β1-mediated SMAD signaling in fibroblasts reinforcing fibrotic cells in a chronically activated state (123, 124). IL-6 inhibits structural repair by interacting with TNF-α and Dickkopf-related protein 1 (DKK-1) to disrupt the Wnt/β-catenin pathway in fibroblasts and in synoviocytes. Th17- and Tγδ17-derived IL-17 activates synoviocytes via the NF-κB and PI3K/AKT pathways inducing IL-6 and IL-8 production, establishing a deleterious feedback loop of T cell-derived IL-17 chronic inflammation (125, 126). A study demonstrated that upregulated IL-17 expression in RA-FLS enhances Bcl-2 expression in RA, promoting cell survival via the JAK/STAT3 pathway, where STA-21, a STAT3 inhibitor, reverses this effect, restoring apoptotic signaling (127). Another study found that IL-17A and TNF synergistically drive inflammation in human synovial fibroblasts and HEK293 cells via the transcription factor ELF3 driving NOS2, PTGS2, and MMP13 expression, promoting tissue destruction in chronic arthritis. While IL-17A or TNF alone weakly induced ELF3, their combination triggered a robust and sustained expression comparable to that of IL-1β. This process requires NF-κB signaling and *de novo* protein synthesis, mediated through the stabilization of IκBζ mRNA. Both IκBζ and C/EBPβ are essential for ELF3 transcription, with ELF3 being indispensable for IL-17A–TNF–induced inflammatory cytokines (128). The modulation of key profibrotic factors by interleukins is often context dependent. For instance, the activation of TGF-β, augmented by inflammatory cytokines and inhibited by IL-10, initiates the fibrotic program through the induction and stabilization of ZEB1. The ZEB1 axis serves as a key molecular switch that controls ECM production (129).

IL-4 and IL-13 play context-dependent roles in RA. Elevated IL-4 levels in the serum and bronchoalveolar lavage fluid (BALF) of RA-ILD patients activate the JAK/STAT6 pathway, promoting ECM synthesis (110, 130), while IL-4 induces eosinophilic inflammation, mucus metaplasia, and subepithelial fibrosis. Mechanistically, both cytokines drive M2 macrophage polarization, a process further amplified by IL-6-enhanced IL-4 receptor signaling, increasing TGF-β production, thereby establishing a self-fibrotic loop (132, 133). Conversely, in CIA models and RA tissues, IL-4 and IL-13 exert protective roles within the synovium by downregulating pro-inflammatory pathways, delaying the onset of arthritis, and reducing cytokine production.

RA being a systemic inflammation, often affects extra-articular organs. In patients with RA, 70% exhibit bronchiolitis, pleural manifestations, rheumatoid nodules, and interstitial lung disease (ILD). Among these, rheumatoid arthritis–associated interstitial lung disease (RA-ILD) represents the most clinically significant manifestation, with cohort studies indicating that approximately 7% of patients develop clinically apparent ILD during disease progression (134). A study explored the therapeutic effects of pirfenidone and nintedanib on pulmonary fibrosis in an RA-ILD disease model, in which a bovine collagen II (bCII)-induced mouse model was utilized. Both drugs significantly reduced alveolar wall thickening, inflammatory cell infiltration, and collagen deposition, indicating the attenuation of pulmonary fibrosis. Mechanistically, the drugs inhibited TGFβR2/SMAD3 signaling pathway and downstream JAK2/STAT3 activation resulting in attenuation of lung fibrosis and decreased ECM. Flow cytometry analysis revealed decreased M1 and M2 macrophages in bronchoalveolar lavage fluid of RA-ILD mice indicating anti-fibrotic and anti-inflammatory activity (135). Another similar study performed in a bCII-induced DBA/1 mouse model studied the therapeutic role of baricitinib in attenuating fibrotic remodeling in the lung. Baricitinib, a JAK1/2 inhibitor, significantly reduced fibrotic expressing genes including, α-SMA, collagen I, collagen IV and fibronectin. Mechanistically, inhibition of JAK2/STAT3 axis suppresses pulmonary fibrosis via the modulation of interconnected TGFβ/SMAD3 pathway (136). Usual interstitial pneumonia (UIP) pattern of lung injury, a key feature of idiopathic pulmonary fibrosis (IPF), is reported in up to 40% of individuals with RA-related ILD (137). This pathological similarity raises the possibility of shared fibrotic pathways. Supporting this notion, a recent study demonstrated that upregulation of SPP1 expression promotes M2 macrophage polarization, enhancing profibrotic mediators, and accelerating EMT and collagen production in IPF, suggesting that similar mechanisms could hypothetically contribute to RA-ILD progression (138).

Although liver injury is not a major pathological feature of RA, studies have reported that 18%–50% of patients with RA have abnormal liver function and elevated alkaline phosphatase levels. A study investigated hepatic fibrosis in a combined CIA and NAFLD animal model and mechanistically identified polymerase I and transcript release factor (PTRF) as key regulators of liver injury and fibrosis. PTRF expression was strongly co-localized with TLR4 and associated with reduced phosphorylation of the PI3/AKT axis. PTRF was mainly localized around small vessels and co-expressed with CD31 and α-SMA, indicating the involvement of endothelial cells and activated hepatic stellate cells in fibrogenesis. Knockdown of PTRF using AAV-mediated shRNA reduced TLR4 signaling, restored PI3K/AKT activation, and significantly alleviated hepatic fibrosis (139). Interestingly, studies in other liver fibrosis models have shown that senescence of activated hepatic stellate cells can act as an intrinsic antifibrotic mechanism. For instance, in CCL4-induced liver injury, p53-dependent stellate cell senescence limits fibrotic progression, whereas p53 deficiency leads to persistent stellate cell activation and enhanced TGF-β1-mediated fibrosis (140). Although this mechanism has not been specifically demonstrated in RA-associated liver injury, these findings raise the possibility that impaired stellate cell senescence may hypothetically contribute to sustained fibrogenesis in conditions associated with RA.

### 5.3 Sjögren’s disease

**Figure 4:**
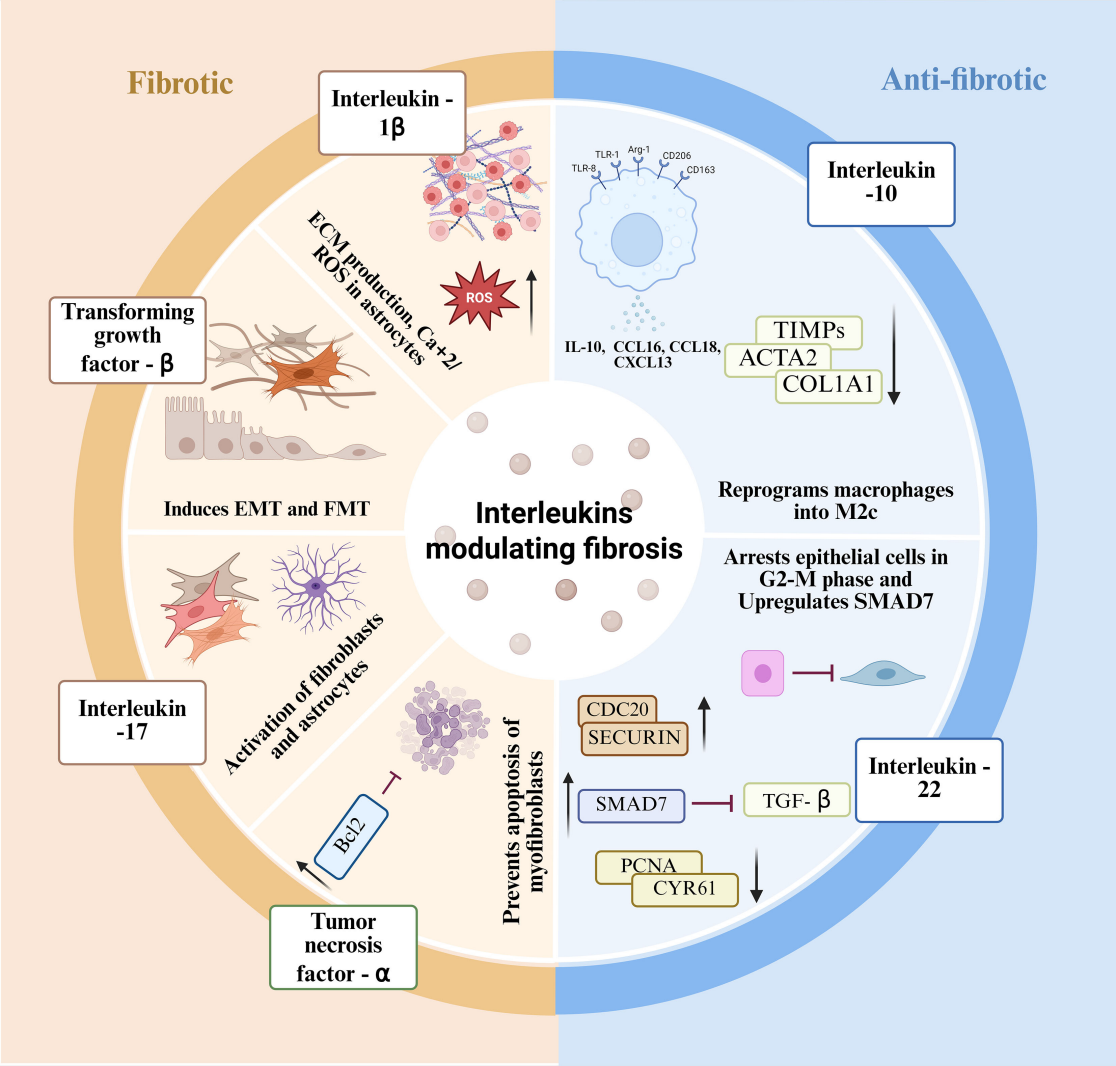
Interleukins modulating Pro- and Anti-fibrotic pathways. (Left) Pro-fibrotic: The fibrotic side highlights the profibrotic cytokines, including IL-1β, IL-17, and TNF-α promotes EMT, fibroblast activation, ROS generation, myofibroblast survival, and ECM deposition. (Right) Anti-fibrotic: The anti-fibrotic side highlights cytokines that play a protective role, includes IL-10, and IL-22 which suppressed ECM protein expression, increase TIMPs, and inhibit inflammatory chemokines. They also reprogram macrophages towards the M2c phenotype and arrest epithelial cells in the G2-M phase. IL-1β, Interleukin-1β; IL-10, Interleukin-10; IL-17, Interleukin-17; IL-22, Interleukin-22; TGF-β, Transforming growth factor-β; TNF-α, Tumor necrosis factor-α; EMT, Epithelial-to-mesenchymal transition; FMT, Fibroblast-to-myofibroblast transition; ROS, Reactive oxygen species; ECM, Extracellular matrix; TIMP, Tissue inhibitor of metalloproteinases; ACTA2, Alpha-smooth muscle actin; COL1A1, Collagen type I alpha 1 chain; SMAD7, Mothers against decapentaplegic homolog 7; CDC20, Cell division cycle protein 20; PCNA, Proliferating cell nuclear antigen; CYR61, Cysteine-rich angiogenic inducer 61; CCL2, C-C motif chemokine ligand 2; CCL5, C-C motif chemokine ligand 5; CCL18, C-C motif chemokine ligand 18; CXCL13, C-X-C motif chemokine ligand 13.

Sjögren’s disease (SjD) involves chronic epithelial damage, leading to persistent inflammation resulting in irreversible fibrosis. The accumulation of mononuclear infiltrates around ducts via the replacement of functional acinar units results in glandular atrophy, architectural distortion, and long-term loss of secretory function (141). Interleukins, such as IL-6, IL-12, IL-18, and IL-1β, along with B-cell activating factor (BAFF), play a pathological role in the development of SjD. IL-17A and IL-17F levels are elevated in both the salivary glands (SG) and serum of patients with primary SjD, where they initiate EMT-dependent fibrosis (142, 143). Recent study employed an experimental Sjögren’s disease (ESjD) mouse model to reveal that non-hematopoietic IL-17 signaling is essential for salivary dysfunction. Mechanistically, IL-17 stabilizes IκB-ζ mRNA and attenuates NF-κB transactivation at the TRPC1 promoter, diminishing TRPC1 expression and impairing cholinergic Ca2+ entry, thereby reducing salivary secretion. Additionally, IL-17-driven epithelial injury promotes EMT and amplifies IL-6/TGF-β signaling, activating stroma and fibroblasts and linking epithelial stress to fibrosis (144). Under mild inflammatory conditions, IL-22 facilitates epithelial survival, and tissue repair; however, in the presence of Type I interferons (IFNs) or IL-17, IL-22’s function shifts towards promoting epithelial stress, apoptosis, and fibrosis. The presence of Type I IFN and IL-17 in NOD mice reprograms IL-22’s protective role against pathogenic epithelial stress, releasing IL-18 and IFNγ. Additionally, IL-22-producing B cells contribute to autoantibody production, collectively establishing fibrotic microenvironment (145). In contrast, another study revealed that IL-22 arrests epithelial proliferation at the G2-M phase checkpoint through the upregulation of CDC20, SECURIN, and CDKN1C, and the downregulation of PCNA, CYR61, and CTGF. It also induces antimicrobial peptides and suppresses IL-8, TGF-β, IL-1α/β, and MMP9, thereby limiting fibrosis. These findings underscore the bidirectional role of IL-22 in SjD (146). In addition to IL-17 and IL-22, IL-13 is a critical fibrotic marker. In Id3-/- mice, IL-13 levels were elevated in the serum, IL-13+ CD4+ T cells, and thymus. Neutralization of IL-13 restored salivary function and reduced mast cell numbers without affecting lymphocytic infiltration, indicating its effect on stromal remodeling rather than immune cell recruitment. IL-13 leads to fibroblast activation, ECM deposition, and tissue remodeling while enhancing mast cell proliferation and FcϵRI upregulation, collectively contributing to fibrosis-like tissue remodeling (147) (**Figure 4****).** A recent mechanistic study investigating intracellular regulators of glandular fibrosis showed an elevated expression of G protein-coupled receptor kinase 2 (GRK2) contributing to fibrotic progression via interaction with TGF-β/Smad2/3. A hemizygous knockout of GRK2 in salivary gland epithelial cells (SGECs) attenuated collagen I production and fibrosis by hindering Smad2/3 nuclear translocation (148). Complementing these findings, experimental studies of epithelial remodeling demonstrated that mesenchymal stem cell transplantation in NOD Sjögren’s mouse models suppress glandular fibrosis by modulation of immune checkpoint inhibitor Tim-3, which is significantly upregulated in submandibular gland of SjD patients. Additionally, MSCs attenuated salivary gland fibrosis by inhibiting EMT marked by a reduction of vimentin and TGF-β, through downregulation of Tim-3 expression in submandibular gland (149).

Additional studies have investigated the role of stromal–immune interactions and the contribution of mast cells. Mast-fibroblast coculture experiment demonstrated that mast cells release TGF-β1, which through stimulation of fibroblasts promote collagen 1 expression (150). More recently, salivary extracellular vesicles (sEVs) have been found to attenuate the core pathways that drive salivary gland fibrosis. This is facilitated by the inhibition of TGF-β-mediated myofibroblast activation via inhibition of STAT3 and its nuclear translocation, leading to the downregulation of fibrotic markers (151). Furthermore, recent evidence has highlighted the relevance of chronic endoplasmic reticulum stress response, marked by elevated BIP and PERK expression together with reduced ATF- 6 and CHOP expression, suggesting chronic, unresolved UPR activation, that favors epithelial survival and long-term inflammation, further perpetuating a chronic ERS status that leads to glandular dysfunction (152). Another similar study evaluated the prevalence of fibrosing patterns in patients with primary Sjögren’s disease–associated interstitial lung disease (SjD-ILD). Among 34 pSjD-ILD patients, 52.9% exhibited a fibrosing pattern, indicating that pulmonary fibrosis is a frequent manifestation of the disease. Interestingly, patients with fibrotic ILD were generally younger and had a shorter disease duration at ILD diagnosis, suggesting that lung involvement may occur during the early phases of disease progression, rather than developing only as a consequence of late-stage pathological complications (153). Liang et al. established a model of SjD-ILD, in which they utilized bleomycin-induced NOD/LtJ mice. Lung tissues exhibited increased collagen deposition, higher Ashcroft fibrosis scores, and elevated expression of α-SMA and collagen-I, indicating extracellular matrix (ECM) deposition and fibrosis. The presence of B-cell-dominant lymphocytic and tertiary lymphoid aggregates was also observed in lung tissues, highlighting the role of adaptive immune responses in SjD-ILD pathogenesis (154).

### 5.4 Crohn’s disease

**Figure 5:**
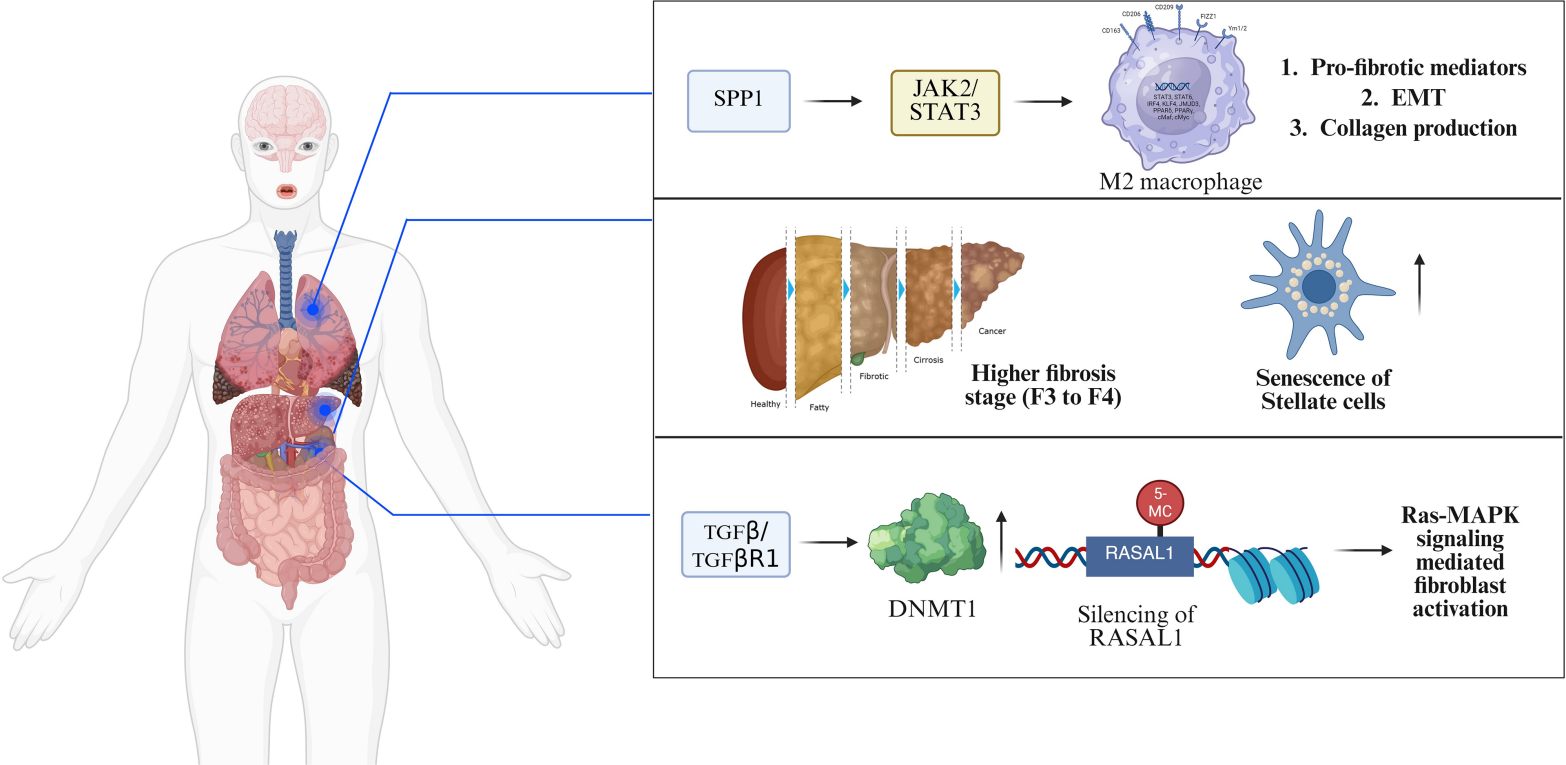
Mechanistic involvement underlying fibrosis in multi-organs. (First) in pulmonary fibrosis, the transcription factor STAT3 promotes M2 macrophage polarization and drives profibrotic mediators, EMT, and increases collagen production via SPP1. (Second)Chronic liver injury induces activation and senescence of stellate cells, contributing to hepatofibrosis (stages F0–F4). (Third)Activation of TGF-β/TGF-βR signaling silences transcriptional upregulation of RASAL1 via DNMT1-mediated epigenetic modification. Subsequent RAS–MAPK activation leads to fibroblast activation and sustained fibrogenic activity. ERK, Extracellular signal-regulated kinase; TGF-β, transforming growth factor-β; TGF-βR, transforming growth factor-β receptor; RASAL1, RAS protein activator like 1; DNMT1, DNA methyltransferase 1; RAS–MAPK, RAS–mitogen-activated protein kinase; STAT3, Signal transducer and activator of transcription 3; EMT, Epithelial-to-mesenchymal transition.

Crohn’s disease (CD), a form of inflammatory bowel disease (IBD), involves chronic intestinal inflammation that drives fibrosis and stricture formation through excessive ECM deposition (155). While interleukins normally aid in mucosal repair, chronic inflammation turns them pathogenic, promoting fibroblast activation, myofibroblast differentiation, and ECM accumulation (156). One of the major log-term complications of Crohn’s disease is the development of intestinal fibrosis, affecting approximately 70% of CD patients after 10 years of diagnosis (157). Intestinal fibrosis in Crohn’s disease is largely mediated by FAP+ myofibroblasts, sustained by IL-17 and IL-6, and perpetuated by inflammatory molecules. This is compounded by the dysregulated activation of mesenchymal cells, coupled with an imbalance between MMPs and TIMPs production (158). This inflammatory state prompts the mobilization of tissue fibroblasts, which, under the influence of TGF-β, differentiate into myofibroblasts (FMT), resulting in the deposition of ECM components (159). Recent studies highlighting the overactivation of MerTK/ERK/TGF-β1 signaling axis has been identified as a crucial driver of intestinal fibrosis. This is facilitated by epithelial cell apoptosis, resulting in the activation of fibroblasts which increased TGF-β secretion. Further, it is amplified by Osteopontin (OPN) through alteration of ERK1/2 phosphorylation. Pharmacological inhibition of MerTK significantly reduced fibrogenic gene expression, collagen deposition and intestinal wall thickening in experimental models (160).Additionally, the interaction between macrophages and T cells, mediated by Th1 cell cytokines (IFN-γ and IL-12), leads to the production of TNF-α, which subsequently upregulates myofibroblast synthesis via TGF-β1 signaling, inhibits MMPs, and promotes TIMPs, leading to uncontrolled collagen deposition (158). Overexpression of IL-17A in Crohn’s disease strictures enhances the production of collagen and TIMP1 by myofibroblasts (161). Another study investigating IL-17A expression found that elevated levels of this cytokine induced EMT, characterized by a reduction in E-cadherin and increased expression of vimentin and α-SMA. This was corroborated by the administration of anti-IL-17 treatment, which resulted in a reduction in the EMT process, thereby decreasing fibrosis (162). Amphiregulin (AREG), a ligand of epidermal growth factor receptor (EGFR), has emerged as a crucial mediator marked by its elevated levels in the fibrotic intestinal tissues, which promote fibroblast activation and ECM deposition. TGF-β – induced Tregs have been identified as a major source of AREG that enhance myofibroblast proliferation, migration, and collagen production through activation of Smad3 confirmed by the presence of α-SMA expression indicative of pathologic fibroblasts. Experimental models further demonstrate that AREG deficiency reduced intestinal fibrosis marked by decreased expression of Col1a1 and Col6a3 despite increased inflammatory activity (163). Complementing these findings, another study showcases that monocytes represent a major cellular source of AREG in stenotic intestinal tissues of CD patients, that enhance fibroblast proliferation, activation and collagen synthesis. Single-cell RNA sequencing (scRNA-seq) revealed elevated AREG expression in inflammatory monocytes (Ly6c^hi^ monocytes) along with PI3K/AKT signaling as the key downstream pathway involved in AREG-mediated fibrotic responses. Functionally, exogenous AREG administration exacerbated collagen deposition and fibrosis in Dextran sulfate sodium (DSS)-induced colitis models (164) Consequently, pathological remodeling and stricture formation in Crohn’s disease occurs as a result of an inflammatory cascade that activates myofibroblasts, leading to dysregulated ECM deposition (**Figure 5**).

## 6 Current therapeutic strategies in fibrosis management

Fibrosis is a pathological condition characterized by the abnormal accumulation of ECM components, primarily collagen, resulting in tissue scarring and eventual organ failure (3). The principal factor contributing to the progression of this condition is the dysregulation and excessive activation of the TGF-β signaling pathway. This pathway facilitates fibroblast proliferation and differentiation into contractile myofibroblasts, while simultaneously increasing ECM synthesis through both canonical (Smad2/3-mediated) and non-canonical (MAPK, PI3K/Akt, and Rho/ROCK) pathways (165). Consequently, it is imperative to develop therapeutic strategies targeting this signaling pathway, mainly focusing on inhibiting SMAD phosphorylation and downregulating the expression of profibrotic genes.

Pharmacological agents have been employed to target the principal pathways and mechanisms implicated in fibrosis, including oxidative stress, inflammation, and immune activation. In addition to addressing these common denominators, certain tyrosine kinase inhibitors have been utilized as effective anti-fibrotic agents, primarily functioning to downregulate fibroblast proliferation. An alternative strategy involves targeting immune dysregulation to mitigate persistent inflammation, which is a predominant feature of fibrosis (166). Lymphocytes exert protective effects against skin fibrosis (167). Elevated oxidative stress levels also serve as critical mediators of this condition, leading to cellular injury and upregulation of the TGF-β signaling pathway (168). This aspect has been explored in patients with renal fibrosis. A list of major therapeutic drugs currently used for the treatment of fibrosis is provided in **Table 2**.

**Table 2:** Summary of target – specific fibrosis inhibitors: selective inhibitors, monoclonal antibodies, immunosuppressive therapy, pre-clinical developments.

Drug	Target	Mode of action	Reference
Selective Inhibitors (Small Molecules)			
Perfenidone	Supresses TGF-β signalling	Antifibrotic, anti-inflammatory and antioxidant	(169)
Nintedanib	Tyrosine kinase inhibitor (PDGF, FGF, VEGF receptors etc.)	Antifibrotic, anti-inflammatory	(170)
Bardoxolone methyl	Activates NRF2	Anti-inflammatory and antioxidant	(171)
Galunisertib	Inhibition of TGF-β receptor type I kinase activation	Antifibrotic, reduces Type I, III, IV, V, collagen	(172)
Immunosuppressive Therapy			
Mycophenolate mofetil	Inhibits proliferation of Lymphocytes	Anti-proliferative	(173)
Methotrexate	Reduces immune cell proliferation	Anti-inflammatory and immunosuppressive	(174)
Monoclonal Antibodies			
Rituximab	Depletion of B lymphocytes	Anti-inflammatory	(175)
FG-3019	Inhibition of CTGF (induced by TGF-β1) and EMT	Anti-fibrotic	(176)
HL217	Targets protein ENO1	Anti- inflammatory, Prevents collagen deposition and lung scarring	(177)
RAGE antibody	Inhibits RAGE pathway	Suppresses activation of HSCs, inhibits expression of fibrosis-associated genes, including COL1A1, TIMP1	(178)
Pre-clinical / Experimental Compounds			
Fluorofenidone	Inhibits TGF-β1/Smad pathway	Reduced ECM synthesis	(179)
Setanaxib	NOX1/NOX4 dual inhibitor	Reduces oxidative stress and myofibroblast activation	(180)
Belapectin	Inhibitor of galactosin-3	Prevents collagen deposition	(181)
Cenicriviroc	Inhibits CCR2 and CCR5 receptors	Inhibits collagen deposition, Anti-inflammatory	(182)

Among the selective small-molecule inhibitors, pirfenidone and nintedanib are approved antifibrotic agents for treating pulmonary fibrosis. Pirfenidone inhibits TGF-β signaling and exhibits antifibrotic, anti-inflammatory, and antioxidant properties. Nintedanib, a tyrosine kinase inhibitor targeting PDGF, FGF, and VEGF receptors, mitigates fibroblast proliferation and differentiation, thereby reducing ECM accumulation (169, 170). Bardoxolone methyl offers protection against renal fibrosis through the activation of the NRF2 pathway, which diminishes oxidative stress and inflammation (171). Similarly, galunisertib, a selective TGF-β receptor type I kinase inhibitor, decreases the synthesis of type I, III, IV, and V collagen in liver fibrosis by disrupting the TGF-β/Smad pathway (172).

Immunosuppressive therapies, including mycophenolate mofetil and methotrexate, regulate immune cell proliferation and cytokine production. Mycophenolate is effective in treating systemic sclerosis and skin fibrosis by inhibiting lymphocyte proliferation. Methotrexate provides anti-inflammatory and immunosuppressive effects by suppressing immune cell activity, thereby offering clinical benefits in both cutaneous and systemic fibrotic disorders (173, 174).

The use of monoclonal antibodies represents a targeted strategy for managing fibrosis. Rituximab effectively reduces inflammation and fibrosis driven by autoimmune processes through the depletion of B lymphocytes. FG-3019 (pamrevlumab) specifically targets connective tissue growth factor (CTGF), a downstream effector of TGF-β1, thereby inhibiting EMT and resulting in a reduction of renal fibrosis (176). HL217, an antibody directed against ENO1, mitigates collagen deposition and lung scarring in cases of pulmonary fibrosis (177). Furthermore, antibodies against the receptor for advanced glycation end-products (RAGE) impede the RAGE pathway, thereby suppressing the activation of hepatic stellate cells (HSC) and downregulating fibrogenic genes such as COL1A1 and TIMP1 (178).

In preclinical and experimental phases, compounds such as fluorofenidone and setanaxib have demonstrated promising efficacy in mitigating fibrosis. AKF-PD inhibits the TGF-β1/Smad pathway, thereby reducing ECM synthesis during liver fibrosis. Setanaxib, a dual NOX1/NOX4 inhibitor, decreases oxidative stress and myofibroblast activation across multiple organs (179, 180). Belapectin, a galectin-3 inhibitor (181), and cenicriviroc, a CCR2/CCR5 antagonist, target inflammatory and fibrogenic signaling pathways, preventing collagen deposition and the progression of fibrosis in liver diseases (183).

Nevertheless, a significant limitation of these strategies is their inability to completely reverse fibrotic conditions, as they primarily aim to halt or decelerate progression rather than achieve permanent cessation. Consequently, the future direction of anti-fibrotic research should transition from disease stabilization to the pursuit of genuine therapeutic reversals. This involves focusing on mechanisms that actively neutralize persistent pro-fibrotic transcriptional drivers, thereby enhancing the likelihood of organ recovery.

## 7 Future perspective

### 7.1 Strategy #1 spatiotemporal cytokine tuning

As previously mentioned, IL-1β and IL-10 exhibit both reparative and pathogenic functions, with IL-1β being essential in the early stages of remyelination and IL-10 offering acute protection but becoming profibrotic when sustained. Therapeutic interventions require precise temporal and spatial modulation. The application of drug delivery systems, such as biodegradable hydrogels and targeted nanoparticles, during the injurious phase, alongside the provision of transient IL-1β during acute repair to enhance the IGF-1/OPC response, is more effective in mitigating fibrosis progression (99). Beyond the chronic systemic treatment window, a comprehensive understanding of the optimal timing for inhibition and enhancement is crucial to maximize therapeutic efficacy and minimize scar tissue formation.

### 7.2 Strategy #2 block feed-forward amplification loops (IL-6/IL-17/TGF-β)

In the context of autoimmune disorders, the disruption of self-amplification loops, such as the IL-17/IL-6-mediated upregulation of TGF-β signaling and the reciprocal enhancement of IL-6/CTGF-driven FMT/EMT by TGF-β, can be achieved using dual inhibitors (e.g., IL-6 trans-signaling inhibitors combined with TGF-β/ALK5 modulators) (183) or small molecule inhibitors. This approach aims to decouple the signaling interactions between inflammatory pathways, such as IL-6/STAT3 and fibrotic pathways, such as TGF-β/SMAD, with the objective of disrupting the fibrotic circuit in conditions such as RA-ILD, pulmonary fibrosis, and MS.

### 7.3 Strategy #3 immune-stromal reprogramming

To alter the phenotype of critical immune cells, specific reprogramming of M2/IL-10-driven macrophages and pathogenic fibroblasts, including EPF, FAPα+, and RASAL1-methylated fibroblasts, can alleviate fibrosis by using transient inhibitors targeting macrophage signals, such as CCR2/CCR5 or galectin-3 (184, 185). Therapeutic strategies should focus on the reversible reprogramming of fibroblasts by reducing α-SMA and collagen levels, while preserving their reparative functions. Interventional methods, such as the inhibition of JAK/STAT6 through modulation of IL-4/IL-13 and restoration of mRNA, including miR-29/miR-30, are instrumental in modulating the progression of fibrosis.

### 7.4 Strategy #4 microenvironment normalization: target NETosis, mechanotransduction, metabolism and senescence

**Figure 6:**
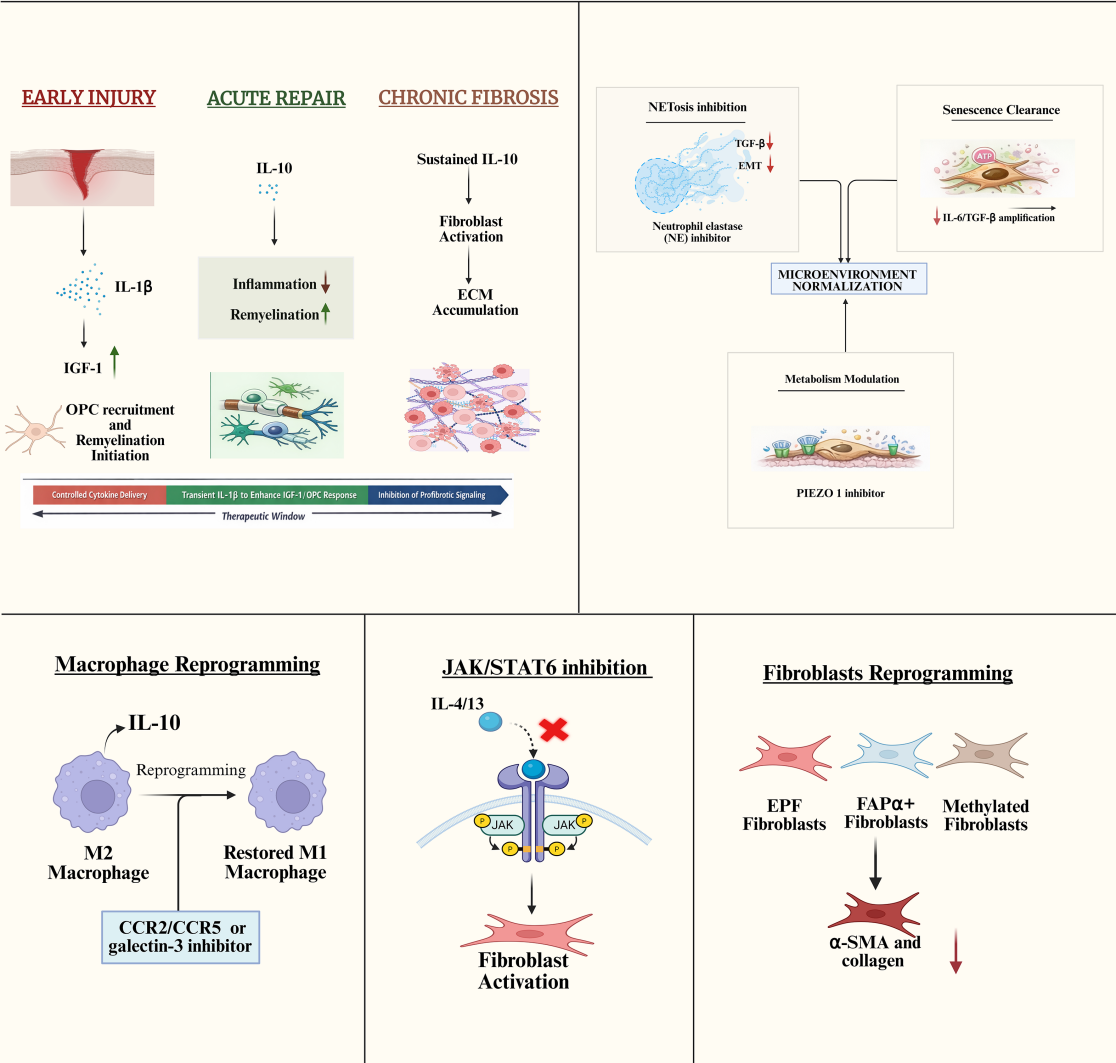
Mechanism-based therapeutic targets for fibrosis resolution. (Top left) Early post injury, IL-1β drives IGF-1–mediated OPC recruitment and remyelination, while transient IL-10 suppresses inflammation. Conversely, sustained IL-10 signaling triggers fibroblast activation and ECM accumulation, leading to chronic fibrosis. Controlled cytokine delivery using biomaterials or nanoparticles enables precise timing of pro-repair and anti-fibrotic signals. (Top right) Targeting non-cytokine drivers such as NET formation, neutrophil elastase activity, senescent cell accumulation, and mechanotransduction pathways (e.g., PIEZO1) can reduce TGF-β activation, EMT, and IL-6/TGF-β amplification. (Bottom) Emerging therapies aim to reprogram M2/IL-10–driven macrophages toward a restorative phenotype via CCR2/CCR5 or galectin-3 blockade, disrupting IL-4/IL-13/JAK/STAT6 axis in fibroblasts, and reverse pathogenic fibroblast states (EPF, FAPα^+^, and methylated fibroblasts) to diminish α-SMA and collagen deposition while preserving reparative functions. IL-1β, Interleukin-1 beta; IGF-1, Insulin growth factor 1; OPC, Oligodendrocyte progenitor cell; IL-10, Interleukin 10; ECM, Extracellular matrix; NET, Neutrophil extracellular trap; PIEZO1, Piezo-type mechanosensitive ion channel component 1; TGF-β, Transforming growth factor beta; EMT, Epithelial-mesenchymal transition; IL-6, Interleukin 6; CCR2, C-C motif chemokine receptor 2; CCR5, C-C motif chemokine receptor 5; IL-4, Interleukin 4; IL-13, Interleukin 13; JAK, Janus kinase; STAT6, Signal transducer and activator of transcription 3; EPF, Engrailed-1 positive fibroblasts; FAPα^+^, Fibroblast activation protein-alpha positive; α-SMA, Alpha smooth muscle actin.

It is imperative to address non-cytokine factors that transform inflammation into an irreversible scarring. Inhibiting neutrophil-driven activation, such as NETosis and neutrophil elastase (NE) activation, can prevent the early activation of TGF-β and EMT (186). Furthermore, modulating mechanosensors, such as PIEZO1 (187), to inhibit fibroblast expansion and eliminate pathological senescent cells to reduce SASP-mediated IL-6/TGF-β amplification is crucial (188). Collectively, these interventions can restore the regenerative niche and alleviate the cytokine pressure that sustains fibrosis (**Figure 6**).

## 8 Existing challenges

Current strategies for addressing fibrosis encounter significant challenges related to biological complexity and clinical implementation. The intrinsic functional duality of cytokines, such as IL-6 and IL-17, presents a critical obstacle, as it complicates the inhibition of pathological processes without simultaneously impairing wound repair mechanisms. Additionally, patient and tissue heterogeneity further complicate therapeutic responses, and the absence of organ-specific delivery mechanisms poses a substantial risk for systemic immunosuppression. Although emerging technologies, such as miRNA replacement and mechanotransduction targets like PIEZO1, offer therapeutic promise, they also raise safety concerns regarding off-target effects and their impact on multiple cell types. Successfully navigating these challenges necessitates dedicated efforts to achieve targeted delivery and develop improved mechanistic biomarkers.

## 9 Conclusion

Fibrosis, characterized by pathological scarring in autoimmune and chronic inflammatory diseases, arises through intricate and interconnected pathways. These pathways encompass molecular dysregulation involving epigenetic fixation, metabolic rewiring, mechanotransduction and cytokine imbalance. Regulatory interleukins (IL-6, IL-17, IL-10, and TGF-β) exert context-dependent and often antagonistic effects that influence tissue progression towards either pathological scarring or repair. Recent discoveries concerning fibroblast lineage identity, macrophage polarization, and epithelial dysregulation have identified crucial therapeutic targets. These findings underscore the urgent need for precise strategies that integrate temporal cytokine modulation and targeted cellular reprogramming to prevent irreversible extracellular matrix accumulation and promote tissue regeneration.

## Glossary

α-SMA: Alpha-Smooth Muscle Actin
AKT: Protein Kinase B
AREG: Amphiregulin
AT2: Alveolar Epithelial Type 2
BALF: Bronchoalveolar Lavage Fluid
BCL-2: B-Cell Lymphoma 2
BL: Bleomycin
BMP: Bone Morphogenetic Protein
CD4+: Clusters of Differentiation 4
CKD: Chronic Kidney Disease
CNS: Central Nervous System
CTGF: Connective Tissue Growth Factor
CXCL: C-X-C Chemokine Ligand
CXCR: C-X-C Motif Chemokine Receptor
CYR61: Cysteine-Rich Protein 61
DKK1: Dickkopf-1
DNMT: DNA Methyltransferase
DSS: Dextran Sulfate Sodium
ECM: Extracellular Matrix
EMT: Epithelial–Mesenchymal Transition
ERK: Extracellular Signal-Regulated Kinase
ESS: Experimental Sjögren’s Syndrome
FGF: Fibroblast Growth Factor
FLS: Fibroblast-Like Synoviocytes
FMT: Fibroblast–Myofibroblast Transition
FOXP3: Forkhead Box P3
GDF: Growth and Differentiation Factors
GRB2: Growth Factor Receptor-Bound Protein 2
HEK293: Human Embryonic Kidney 293
IBD: Inflammatory Bowel Disease
IFN-γ: Interferon Gamma
IKK: Inhibitor of κB Kinase
ILD: Interstitial Lung Disease
JAK/STAT: Janus Kinase/Signal Transducer and Activator of Transcription
LAP: Latency-Associated Peptide
LSEC: Liver Sinusoidal Endothelial Cells
LTBP: Latent TGF-β
MAPK: Mitogen-Activated Protein Kinase
MIOX: Myo-Inositol Oxygenase
MIP-1α: Macrophage Inflammatory Protein-1 Alpha
MMPs: Matrix Metalloproteinases
MS: Multiple Sclerosis
mTOR: Mammalian Target of Rapamycin
NF-κB: Nuclear Factor Kappa-Light-Chain Enhancer of Activated B Cells
NO: Nitric Oxide
NOS2: Nitric Oxide Synthase 2
OPC: Oligodendrocyte Progenitor Cells
PCNA: Proliferating Cell Nuclear Antigen
PDGF: Platelet-Derived Growth Factor
PI3K: Phosphoinositide 3-Kinase
PTGS2: Prostaglandin-Endoperoxide Synthase 2
RAG1: Recombination Activating Gene 1
RAS: Rat Sarcoma Virus
RASAL1: RAS Protein Activator Like 1
RORγt: Retinoic Acid-Related Orphan Receptor Gamma t
ROS: Reactive Oxygen Species
SG: Salivary Gland
SHC: Src Homology 2 Domain-Containing Transforming Protein 1
SMAD: Mothers Against Decapentaplegic Homolog
SOS: Son of Sevenless
SPP1: Secreted Phosphoprotein 1
TGF-β: Transforming Growth Factor Beta
TGFR: Transforming Growth Factor Beta Receptor
THP-1: Tohoku Hospital Pediatrics-1
TIMPs: Tissue Inhibitors of Metalloproteinases
TNF-α: Tumor Necrosis Factor Alpha
TRAF6: Tumor Necrosis Factor Receptor–Associated Factor 6
TRPC1: Transient Receptor Potential Cation Channel Subfamily C Member 1
UUO: Unilateral Ureteral Obstruction
VEGF: Vascular Endothelial Growth Factor
WNT: Wingless-Related Integration Site
ZO-1: Zonula Occludens-1.

## References

[1] Lurje I Gaisa NT Weiskirchen R Tacke F . Mechanisms of organ fibrosis: Emerging concepts and implications for novel treatment strategies. Mol Aspects Med. (2023) 92. doi: 10.1016/j.mam.2023.101191. PMID: 37236017

[2] Żurawek M Ziółkowska-Suchanek I Iżykowska K . Fibrosis in immune-mediated and autoimmune disorders. J Clin Med. (2025) 14:6636. doi: 10.3390/jcm14186636. PMID: 41010839 PMC12470461

[3] Wynn TA Ramalingam TR . Mechanisms of fibrosis: Therapeutic translation for fibrotic disease. Nat Med. (2012) 18:1028–40. doi: 10.1038/nm.2807. PMID: 22772564 PMC3405917

[4] Horowitz JC Thannickal VJ . Mechanisms for the resolution of organ fibrosis. Physiology. (2019) 34:43–55. doi: 10.1152/physiol.00033.2018. PMID: 30540232 PMC6383633

[5] Wynn T . Cellular and molecular mechanisms of fibrosis. J Pathol. (2008) 214:199–210. doi: 10.1002/path.2277. PMID: 18161745 PMC2693329

[6] Froom ZSCS Callaghan NI Davenport Huyer L . Cellular crosstalk in fibrosis: Insights into macrophage and fibroblast dynamics. J Biol Chem. (2025) 301:110203. doi: 10.1016/j.jbc.2025.110203. PMID: 40334985 PMC12167814

[7] Talbott HE Mascharak S Griffin M Wan DC Longaker MT . Wound healing, fibroblast heterogeneity, and fibrosis. Cell Stem Cell. (2022) 29:1161–80. doi: 10.1016/j.stem.2022.07.006. PMID: 35931028 PMC9357250

[8] Zhao M Wang L Wang M Zhou S Lu Y Cui H . Targeting fibrosis, mechanisms and clinical trials. Signal Transduct Target Ther. (2022) 7. doi: 10.1038/s41392-022-01070-3. PMID: 35773269 PMC9247101

[9] Dees C Pötter S Zhang Y Bergmann C Zhou X Luber M . TGF-β–induced epigenetic deregulation of SOCS3 facilitates STAT3 signaling to promote fibrosis. J Clin Invest. (2020) 130:2347–63. doi: 10.1172/JCI122462. PMID: 31990678 PMC7190914

[10] Zhang H-Y Phan SH . Inhibition of myofibroblast apoptosis by transforming growth factor β _1_. Am J Respir Cell Mol Biol. (1999) 21:658–65. doi: 10.1165/ajrcmb.21.6.3720. PMID: 10572062

[11] Merkt W Zhou Y Han H Lagares D . Myofibroblast fate plasticity in tissue repair and fibrosis: Deactivation, apoptosis, senescence and reprogramming. Wound Repair Regener. (2021) 29:678–91. doi: 10.1111/wrr.12952. PMID: 34117675 PMC12854804

[12] Landén NX Li D Ståhle M . Transition from inflammation to proliferation: a critical step during wound healing. Cell Mol Life Sci. (2016) 73:3861–85. doi: 10.1007/s00018-016-2268-0. PMID: 27180275 PMC5021733

[13] Qian H Deng C Chen S Zhang X He Y Lan J . Targeting pathogenic fibroblast-like synoviocyte subsets in rheumatoid arthritis. Arthritis Res Ther. (2024) 26:103. doi: 10.1186/s13075-024-03343-4. PMID: 38783357 PMC11112866

[14] Madsen SF Madsen SS Madrid AS Andersen MR Bay-Jensen A-C Thudium CS . Fibrotic remodeling in joint diseases: Induction and inhibition of fibrosis in fibroblast-like synoviocytes. Transl Med Commun. (2024) 9. doi: 10.1186/s41231-024-00180-0. PMID: 41863001

[15] Leehan KM Pezant NP Rasmussen A Grundahl K Moore JS Radfar L . Minor salivary gland fibrosis in Sjögren’s syndrome is elevated, associated with focus score and not solely a consequence of aging. Clin Exp Rheumatol. (2018) 36:80–8. PMC5913007 29148407

[16] Ma D Feng Y Lin X . Immune and non-immune mediators in the fibrosis pathogenesis of salivary gland in Sjögren’s syndrome. Front Immunol. (2024) 15:1421436. doi: 10.3389/fimmu.2024.1421436. PMID: 39469708 PMC11513355

[17] Bhattacharyya S Wei J Varga J . Understanding fibrosis in systemic sclerosis: Shifting paradigms, emerging opportunities. Nat Rev Rheumatol. (2012) 8:42–54. doi: 10.1038/nrrheum.2011.149. PMID: 22025123 PMC3954787

[18] Ho YY Lagares D Tager AM Kapoor M . Fibrosis - A lethal component of systemic sclerosis. Nat Rev Rheumatol. (2014) 10:390–402. doi: 10.1038/nrrheum.2014.53. PMID: 24752182

[19] Li C Kuemmerle JF . The fate of myofibroblasts during the development of fibrosis in Crohn’s disease. J Dig Dis. (2020) 21:326–31. doi: 10.1111/1751-2980.12852. PMID: 32092217

[20] Catz SD McLeish KR . Therapeutic targeting of neutrophil exocytosis. J Leukoc Biol. (2020) 107:393–408. doi: 10.1002/JLB.3RI0120-645R. PMID: 31990103 PMC7044074

[21] Takemasa A Ishii Y Fukuda T . A neutrophil elastase inhibitor prevents bleomycin-induced pulmonary fibrosis in mice. Eur Respir J. (2012) 40:1475–82. doi: 10.1183/09031936.00127011. PMID: 22441751

[22] Crowley LE Stockley RA Thickett DR Dosanjh D Scott A Parekh D . Neutrophil dynamics in pulmonary fibrosis: Pathophysiological and therapeutic perspectives. Eur Respir Rev. (2024) 33:240139. doi: 10.1183/16000617.0139-2024. PMID: 39603661 PMC11600124

[23] Paish HL Kalson NS Smith GR del Carpio Pons A Baldock TE Smith N . Fibroblasts promote inflammation and pain via IL-1α induction of the monocyte chemoattractant chemokine (C-C motif) ligand 2. Am J Pathol. (2018) 188:696–714. doi: 10.1016/j.ajpath.2017.11.007. PMID: 29248462 PMC5842035

[24] de Almeida LGN Thode H Eslambolchi Y Chopra S Young D Gill S . Matrix metalloproteinases: From molecular mechanisms to physiology, pathophysiology, and pharmacology. Pharmacol Rev. (2022) 74:714–70. doi: 10.1124/pharmrev.121.000349. PMID: 35738680

[25] Jiang Y Cai R Huang Y Zhu L Xiao L Wang C . Macrophages in organ fibrosis: From pathogenesis to therapeutic targets. Cell Death Discov. (2024) 10:487. doi: 10.1038/s41420-024-02247-1. PMID: 39632841 PMC11618518

[26] Jia H Yue G Li P Peng R Jin R Chen Y . Neutrophil extracellular traps license macrophage production of chemokines to facilitate CD8+ T cell infiltration in obstruction-induced renal fibrosis. Protein Cell. (2025) 16 (09):782–798. doi: 10.1093/procel/pwaf020. PMID: 39998389

[27] Kokubo K Onodera A Kiuchi M Tsuji K Hirahara K Nakayama T . Conventional and pathogenic Th2 cells in inflammation, tissue repair, and fibrosis. Front Immunol. (2022) 13:945063. doi: 10.3389/fimmu.2022.945063. PMID: 36016937 PMC9395650

[28] Doucet C Brouty-Boyé D Pottin-Clémenceau C Canonica GW Jasmin C Azzarone B . Interleukin (IL) 4 and IL-13 act on human lung fibroblasts. Implication in asthma. J Clin Invest. (1998) 101:2129–39. doi: 10.1172/JCI741. PMID: 9593769 PMC508801

[29] Belperio JA Dy M Burdick MD Xue YY Li K Elias JA . Interaction of IL-13 and C10 in the pathogenesis of bleomycin-induced pulmonary fibrosis. Am J Respir Cell Mol Biol. (2002) 27:419–27. doi: 10.1165/rcmb.2002-0009OC. PMID: 12356575

[30] Reiman RM Thompson RW Feng CG Hari D Knight R Cheever AW . Interleukin-5 (IL-5) augments the progression of liver fibrosis by regulating IL-13 activity. Infect Immun. (2006) 74:1471–9. doi: 10.1128/IAI.74.3.1471-1479.2006. PMID: 16495517 PMC1418671

[31] Gurujeyalakshmi G Giri SN . MOLECULAR MECHANISMS OF ANTIFIBROTIC EFFECT OF INTERFERON GAMMA IN BLEOMYCIN-MOUSE MODEL OF LUNG FIBROSIS: Downregulation of TGF-P and Procollagen I and 111 Gene Expression For personal use only. (1995) 21:791–808. doi: 10.3109/01902149509050842, PMID: 8556994

[32] Vu TN Chen X Foda HD Smaldone GC Hasaneen NA . Interferon-γ enhances the antifibrotic effects of pirfenidone by attenuating IPF lung fibroblast activation and differentiation. Respir Res. (2019) 20:206. doi: 10.1186/s12931-019-1171-2. PMID: 31511015 PMC6737625

[33] Garibaldi BT D’Alessio FR Mock JR Files DC Chau E Eto Y . Regulatory T cells reduce acute lung injury fibroproliferation by decreasing fibrocyte recruitment. Am J Respir Cell Mol Biol. (2013) 48:35–43. doi: 10.1165/rcmb.2012-0198OC. PMID: 23002097 PMC3547087

[34] Peng X Moore MW Peng H Sun H Gan Y Homer RJ . CD4+CD25+FoxP3+ regulatory Tregs inhibit fibrocyte recruitment and fibrosis via suppression of FGF-9 production in the TGF-Î^2^1 exposed murine lung. Front Pharmacol. (2014) 5:80. doi: 10.3389/fphar.2014.00080. PMID: 24904415 PMC4032896

[35] Yu L Border WA Huang Y Noble NA . TGF-β isoforms in renal fibrogenesis. Kidney Int. (2003) 64:844–56. doi: 10.1046/j.1523-1755.2003.00162.x. PMID: 12911534

[36] Blobe GC Schiemann WP Lodish HF . Role of transforming growth factor β in human disease. N Engl J Med. (2000) 342:1350–8. doi: 10.1056/NEJM200005043421807. PMID: 10793168

[37] Chen W ten Dijke P . Immunoregulation by members of the TGFβ superfamily. Nat Rev Immunol. (2016) 16:723–40. doi: 10.1038/nri.2016.112. PMID: 27885276

[38] MacFarlane EG Haupt J Dietz HC Shore EM . TGF-β family signaling in connective tissue and skeletal diseases. Cold Spring Harb Perspect Biol. (2017) 9:a022269. doi: 10.1101/cshperspect.a022269. PMID: 28246187 PMC5666637

[39] Mallikarjuna P Zhou Y Landström M . The synergistic cooperation between TGF-β and hypoxia in cancer and fibrosis. Biomolecules. (2022) 12:635. doi: 10.3390/biom12050635. PMID: 35625561 PMC9138354

[40] Dinarello CA . Overview of the IL‐1 family in innate inflammation and acquired immunity. Immunol Rev. (2018) 281:8–27. doi: 10.1111/imr.12621. PMID: 29247995 PMC5756628

[41] Itoh Y . Membrane-type matrix metalloproteinases: Their functions and regulations. Matrix Biol. (2015) 44–46:207–23. doi: 10.1016/j.matbio.2015.03.004. PMID: 25794647

[42] Robertson IB Horiguchi M Zilberberg L Dabovic B Hadjiolova K Rifkin DB . Latent TGF-β-binding proteins. Matrix Biol. (2015) 47:44–53. doi: 10.1016/j.matbio.2015.05.005. PMID: 25960419 PMC4844006

[43] Annes JP Munger JS Rifkin DB . Making sense of latent TGFβ activation. J Cell Sci. (2003) 116:217–24. doi: 10.1242/jcs.00229. PMID: 12482908

[44] Yu Q Stamenkovic I . Cell surface-localized matrix metalloproteinase-9 proteolytically activates TGF-β and promotes tumor invasion and angiogenesis. Genes Dev. (2000) 14:163–76. doi: 10.1101/gad.14.2.163. PMID: 10652271 PMC316345

[45] Brooks PC Strömblad S Sanders LC von Schalscha TL Aimes RT Stetler-Stevenson WG . Localization of matrix metalloproteinase MMP-2 to the surface of invasive cells by interaction with integrin αvβ3. Cell. (1996) 85:683–93. doi: 10.1016/S0092-8674(00)81235-0. PMID: 8646777

[46] Zhang YE . Non-Smad pathways in TGF-β signaling. Cell Res. (2009) 19:128–39. doi: 10.1038/cr.2008.328. PMID: 19114990 PMC2635127

[47] Shi Y Massagué J . Mechanisms of TGF-β signaling from cell membrane to the nucleus. Cell. (2003) 113:685–700. doi: 10.1016/S0092-8674(03)00432-X. PMID: 12809600

[48] Lee MK Pardoux C Hall MC Lee PS Warburton D Qing J . TGF-beta activates Erk MAP kinase signaling through direct phosphorylation of ShcA. EMBO J. (2007) 26:3957–67. doi: 10.1038/sj.emboj.7601818. PMID: 17673906 PMC1994119

[49] Deng Z Fan T Xiao C Tian H Zheng Y Li C . TGF-β signaling in health, disease and therapeutics. Signal Transduct Target Ther. (2024) 9:61. doi: 10.1038/s41392-024-01764-w. PMID: 38514615 PMC10958066

[50] Bakin AV Tomlinson AK Bhowmick NA Moses HL Arteaga CL . Phosphatidylinositol 3-kinase function is required for transforming growth factor β-mediated epithelial to mesenchymal transition and cell migration. J Biol Chem. (2000) 275:36803–10. doi: 10.1074/jbc.M005912200. PMID: 10969078

[51] Lamouille S Derynck R . Cell size and invasion in TGF-β–induced epithelial to mesenchymal transition is regulated by activation of the mTOR pathway. J Cell Biol. (2007) 178:437–51. doi: 10.1083/jcb.200611146. PMID: 17646396 PMC2064840

[52] Tomasek JJ Gabbiani G Hinz B Chaponnier C Brown RA . Myofibroblasts and mechano: Regulation of connective tissue remodeling. Nat Rev Mol Cell Biol. (2002) 3:349–63. doi: 10.1038/nrm809. PMID: 11988769

[53] Miettinen LJ Ebner R Lopez AR Derynck R . TGF-/3 induced transdifferentiation of mammary epithelial cells to mesenchymal cells: involvement of type I receptors. 127. doi: 10.1083/jcb.127.6.2021, PMID: 7806579 PMC2120317

[54] Vaughan MB Howard EW Tomasek JJ . Transforming growth factor-β1 promotes the morphological and functional differentiation of the myofibroblast. Exp Cell Res. (2000) 257:180–9. doi: 10.1006/excr.2000.4869. PMID: 10854066

[55] Serini G Gabbiani G . Mechanisms of myofibroblast activity and phenotypic modulation. Exp Cell Res. (1999) 250:273–83. doi: 10.1006/excr.1999.4543. PMID: 10413583

[56] Hinz B Celetta G Tomasek JJ Gabbiani G Chaponnier C . Alpha-smooth muscle actin expression upregulates fibroblast contractile activity. (2001) 12:2730–41. doi: 10.1091/mbc.12.9.2730, PMID: 11553712 PMC59708

[57] Zhou L Ivanov II Spolski R Min R Shenderov K Egawa T . IL-6 programs TH-17 cell differentiation by promoting sequential engagement of the IL-21 and IL-23 pathways. Nat Immunol. (2007) 8:967–74. doi: 10.1038/ni1488. PMID: 17581537

[58] Meng F Wang K Aoyama T Grivennikov SI Paik Y Scholten D . Interleukin-17 signaling in inflammatory, Kupffer cells, and hepatic stellate cells exacerbates liver fibrosis in mice. Gastroenterology. (2012) 143:765–776.e3. doi: 10.1053/j.gastro.2012.05.049. PMID: 22687286 PMC3635475

[59] Simonian PL Roark CL Wehrmann F Lanham AK Diaz del Valle F Born WK . Th17-polarized immune response in a murine model of hypersensitivity pneumonitis and lung fibrosis. J Immunol. (2009) 182:657–65. doi: 10.4049/jimmunol.182.1.657. PMID: 19109199 PMC2766086

[60] Veldhoen M Hocking RJ Flavell RA Stockinger B . Signals mediated by transforming growth factor-β initiate autoimmune encephalomyelitis, but chronic inflammation is needed to sustain disease. Nat Immunol. (2006) 7:1151–6. doi: 10.1038/ni1391. PMID: 16998492

[61] Annunziato F Cosmi L Santarlasci V Maggi L Liotta F Mazzinghi B . Phenotypic and functional features of human Th17 cells. J Exp Med. (2007) 204:1849–61. doi: 10.1084/jem.20070663. PMID: 17635957 PMC2118657

[62] François A Gombault A Villeret B Alsaleh G Fanny M Gasse P . B cell activating factor is central to bleomycin- and IL-17-mediated experimental pulmonary fibrosis. J Autoimmun. (2015) 56:1–11. doi: 10.1016/j.jaut.2014.08.003. PMID: 25441030

[63] Della-Torre E Rigamonti E Perugino C Baghai-Sain S Sun N Kaneko N . B lymphocytes directly contribute to tissue fibrosis in patients with IgG4-related disease. J Allergy Clin Immunol. (2020) 145:968–981.e14. doi: 10.1016/j.jaci.2019.07.004. PMID: 31319101 PMC6960365

[64] Horii M Fushida N Ikeda T Oishi K Hamaguchi Y Ikawa Y . Cytokine‐producing B‐cell balance associates with skin fibrosis in patients with systemic sclerosis. J Dermatol. (2022) 49:1012–9. doi: 10.1111/1346-8138.16495. PMID: 35751840

[65] Hurdayal R Brombacher F . Interleukin-4 receptor alpha: From innate to adaptive immunity in murine models of cutaneous leishmaniasis. Front Immunol. (2017) 8:1354. doi: 10.3389/fimmu.2017.01354. PMID: 29176972 PMC5686050

[66] Bhogal RK Stoica CM McGaha TL Bona CA . Molecular aspects of regulation of collagen gene expression in fibrosis. J ClinImmunol. (2005) 25:592–603. doi: 10.1007/s10875-005-7827-3. PMID: 16380822

[67] Medvedeva GF Kuzmina DO Nuzhina J Shtil AA Dukhinova MS . How macrophages become transcriptionally dysregulated: A hidden impact of antitumor therapy. Int J Mol Sci. (2021) 22:2662. doi: 10.3390/ijms22052662. PMID: 33800829 PMC7961970

[68] Duan Z Luo Y . Targeting macrophages in cancer immunotherapy. Signal Transduct Target Ther. (2021) 6:127. doi: 10.1038/s41392-021-00506-6. PMID: 33767177 PMC7994399

[69] Ueshima E Fujimori M Kodama H Felsen D Chen J Durack JC . Macrophage-secreted TGF-β _1_ contributes to fibroblast activation and ureteral stricture after ablation injury. Am J Physiol Renal Physiol. (2019) 317:F52–64. doi: 10.1152/ajprenal.00260.2018. PMID: 31017012 PMC6692725

[70] Neary R Watson CJ Baugh JA . Epigenetics and the overhealing wound: the role of DNA methylation in fibrosis. Fibrogenesis Tissue Repair. (2015) 8:18. doi: 10.1186/s13069-015-0035-8. PMID: 26435749 PMC4591063

[71] Das PM Singal R . DNA methylation and cancer. J Clin Oncol. (2004) 22:4632–42. doi: 10.1200/JCO.2004.07.151. PMID: 15542813

[72] Hite KC Adams VH Hansen JC . Recent advances in MeCP2 structure and function. This paper is one of a selection of papers published in this Special Issue, entitled 29th Annual International Asilomar Chromatin and Chromosomes Conference, and has undergone the Journal’s usual peer review process. Biochem Cell Biol. (2009) 87:219–27. doi: 10.1139/O08-115. PMID: 19234536 PMC2874317

[73] Mann J Chu DCK Maxwell A Oakley F Zhu N Tsukamoto H . MeCP2 controls an epigenetic pathway that promotes myofibroblast transdifferentiation and fibrosis. Gastroenterology. (2010) 138:705–714.e4. doi: 10.1053/j.gastro.2009.10.002. PMID: 19843474 PMC2819585

[74] Tao H Huang C Yang J-J Ma T-T Bian E-B Zhang L . MeCP2 controls the expression of RASAL1 in the hepatic fibrosis in rats. Toxicology. (2011) 290:327–33. doi: 10.1016/j.tox.2011.10.011. PMID: 22056649

[75] Cedar H Bergman Y . Linking DNA methylation and histone modification: patterns and paradigms. Nat Rev Genet. (2009) 10:295–304. doi: 10.1038/nrg2540. PMID: 19308066

[76] Bechtel W McGoohan S Zeisberg EM Müller GA Kalbacher H Salant DJ . Methylation determines fibroblast activation and fibrogenesis in the kidney. Nat Med. (2010) 16:544–50. doi: 10.1038/nm.2135. PMID: 20418885 PMC3106179

[77] Tao H Yang J-J Hu W Shi K-H Deng Z-Y Li J . MeCP2 regulation of cardiac fibroblast proliferation and fibrosis by down-regulation of DUSP5. Int J Biol Macromol. (2016) 82:68–75. doi: 10.1016/j.ijbiomac.2015.10.076. PMID: 26511729

[78] Xu X Tan X Tampe B Nyamsuren G Liu X Maier LS . Epigenetic balance of aberrant Rasal1 promoter methylation and hydroxymethylation regulates cardiac fibrosis. Cardiovasc Res. (2015) 105:279–91. doi: 10.1093/cvr/cvv015. PMID: 25616414

[79] Kouzarides T . Chromatin modifications and their function. Cell. (2007) 128:693–705. doi: 10.1016/j.cell.2007.02.005. PMID: 17320507

[80] Banerjee T Chakravarti D . A peek into the complex realm of histone phosphorylation. Mol Cell Biol. (2011) 31:4858–73. doi: 10.1128/MCB.05631-11. PMID: 22006017 PMC3233023

[81] O’Reilly S . Epigenetics in fibrosis. Mol Aspects Med. (2017) 54:89–102. doi: 10.1016/j.mam.2016.10.001. PMID: 27720780

[82] Mannaerts I Nuytten NR Rogiers V Vanderkerken K van Grunsven LA Geerts A . Chronic administration of valproic acid inhibits activation of mouse hepatic stellate cells *in vitro* and *in vivo*. Hepatology. (2010) 51:603–14. doi: 10.1002/hep.23334. PMID: 19957378

[83] Elsharkawy AM Oakley F Lin F Packham G Mann DA Mann J . The NF-κB p50:p50:HDAC-1 repressor complex orchestrates transcriptional inhibition of multiple pro-inflammatory genes. J Hepatol. (2010) 53:519–27. doi: 10.1016/j.jhep.2010.03.025. PMID: 20579762 PMC3098379

[84] Qin L Han Y-P . Epigenetic repression of matrix metalloproteinases in myofibroblastic hepatic stellate cells through histone deacetylases 4. Am J Pathol. (2010) 177:1915–28. doi: 10.2353/ajpath.2010.100011. PMID: 20847282 PMC2947286

[85] Kaimori A Potter JJ Choti M Ding Z Mezey E Koteish AA . Histone deacetylase inhibition suppresses the transforming growth factor β1-induced epithelial-to-mesenchymal transition in hepatocytes†,‡. Hepatology. (2010) 52:1033–45. doi: 10.1002/hep.23765. PMID: 20564330

[86] Ulukan B Sila Ozkaya Y Zeybel M . Advances in the epigenetics of fibroblast biology and fibrotic diseases. Curr Opin Pharmacol. (2019) 49:102–9. doi: 10.1016/j.coph.2019.10.001. PMID: 31731224

[87] Ha M Kim VN . Regulation of microRNA biogenesis. Nat Rev Mol Cell Biol. (2014) 15:509–24. doi: 10.1038/nrm3838. PMID: 25027649

[88] Vettori S . Role of microRNAs in fibrosis. Open Rheumatol J. (2012) 6:130–9. doi: 10.2174/1874312901206010130. PMID: 22802911 PMC3396185

[89] van Almen GC Verhesen W van Leeuwen REW van de Vrie M Eurlings C Schellings MWM . MicroRNA‐18 and microRNA‐19 regulate CTGF and TSP‐1 expression in age‐related heart failure. Aging Cell. (2011) 10:769–79. doi: 10.1111/j.1474-9726.2011.00714.x. PMID: 21501375 PMC3193380

[90] Duisters RF Tijsen AJ Schroen B Leenders JJ Lentink V van der Made I . miR-133 and miR-30 regulate connective tissue growth factor. Circ Res. (2009) 104:170–8. doi: 10.1161/CIRCRESAHA.108.182535. PMID: 19096030

[91] Thum T Gross C Fiedler J Fischer T Kissler S Bussen M . MicroRNA-21 contributes to myocardial disease by stimulating MAP kinase signaling in fibroblasts. Nature. (2008) 456:980–4. doi: 10.1038/nature07511. PMID: 19043405

[92] Bian E-B Huang C Ma T-T Tao H Zhang H Cheng C . DNMT1-mediated PTEN hypermethylation confers hepatic stellate cell activation and liver fibrogenesis in rats. Toxicol Appl Pharmacol. (2012) 264:13–22. doi: 10.1016/j.taap.2012.06.022. PMID: 22841775

[93] Tao H Shi P Zhao X Xuan H Gong W Ding X . DNMT1 deregulation of SOCS3 axis drives cardiac fibroblast activation in diabetic cardiac fibrosis. J Cell Physiol. (2021) 236:3481–94. doi: 10.1002/jcp.30078. PMID: 32989761

[94] Tao H Yang J-J Chen Z-W Xu S-S Zhou X Zhan H-Y . DNMT3A silencing RASSF1A promotes cardiac fibrosis through upregulation of ERK1/2. Toxicology. (2014) 323:42–50. doi: 10.1016/j.tox.2014.06.006. PMID: 24945829

[95] Yin S Zhang Q Yang J Lin W Li Y Chen F . TGFβ-incurred epigenetic aberrations of miRNA and DNA methyltransferase suppress Klotho and potentiate renal fibrosis. Biochim Biophys Acta (BBA) - Mol Cell Res. (2017) 1864:1207–16. doi: 10.1016/j.bbamcr.2017.03.002. PMID: 28285987

[96] Pan X You H Wang L Bi Y Yang Y Meng H . Methylation of RCAN1.4 mediated by DNMT1 and DNMT3b enhances hepatic stellate cell activation and liver fibrogenesis through Calcineurin/NFAT3 signaling. Theranostics. (2019) 9:4308–23. doi: 10.7150/thno.32710. PMID: 31285763 PMC6599664

[97] Qu L Wang T Kong J Wu X Li Q Long T . DNMT3B aggravated renal fibrosis in diabetic kidney disease via activating Wnt/β-catenin signaling pathway. Sci Rep. (2025) 15:21070. doi: 10.1038/s41598-025-06713-3. PMID: 40595027 PMC12218845

[98] Liu N He S Ma L Ponnusamy M Tang J Tolbert E . Blocking the class I histone deacetylase ameliorates renal fibrosis and inhibits renal fibroblast activation via modulating TGF-beta and EGFR signaling. PloS One. (2013) 8:e54001. doi: 10.1371/journal.pone.0054001. PMID: 23342059 PMC3546966

[99] Li X Wu X-Q Xu T Li X-F Yang Y Li W-X . Role of histone deacetylases (HDACs) in progression and reversal of liver fibrosis. Toxicol Appl Pharmacol. (2016) 306:58–68. doi: 10.1016/j.taap.2016.07.003. PMID: 27396813

[100] Zheng Q Lei Y Hui S Tong M Liang L . HDAC3 promotes pulmonary fibrosis by activating NOTCH1 and STAT1 signaling and up-regulating inflammasome components AIM2 and ASC. Cytokine. (2022) 153:155842. doi: 10.1016/j.cyto.2022.155842. PMID: 35306425

[101] Yu F Guo Y Chen B Dong P Zheng J . MicroRNA-17-5p activates hepatic stellate cells through targeting of Smad7. Lab Invest. (2015) 95:781–9. doi: 10.1038/labinvest.2015.58. PMID: 25915722

[102] Wang B Koh P Winbanks C Coughlan MT McClelland A Watson A . miR-200a prevents renal fibrogenesis through repression of TGF-β2 expression. Diabetes. (2011) 60:280–7. doi: 10.2337/db10-0892. PMID: 20952520 PMC3012183

[103] Venugopal SK Jiang J Kim T-H Li Y Wang S-S Torok NJ . Liver fibrosis causes downregulation of miRNA-150 and miRNA-194 in hepatic stellate cells, and their overexpression causes decreased stellate cell activation. Am J Physiology-Gastrointestinal Liver Physiol. (2010) 298:G101–6. doi: 10.1152/ajpgi.00220.2009. PMID: 19892940 PMC2806096

[104] Kato M Zhang J Wang M Lanting L Yuan H Rossi JJ . MicroRNA-192 in diabetic kidney glomeruli and its function in TGF-β-induced collagen expression via inhibition of E-box repressors. Proc Natl Acad Sci. (2007) 104:3432–7. doi: 10.1073/pnas.0611192104. PMID: 17360662 PMC1805579

[105] Kriegel AJ Fang Y Liu Y Tian Z Mladinov D Matus IR . MicroRNA-target pairs in human renal epithelial cells treated with transforming growth factor β1: a novel role of miR-382. Nucleic Acids Res. (2010) 38:8338–47. doi: 10.1093/nar/gkq718. PMID: 20716515 PMC3001085

[106] Cho J-H Gelinas R Wang K Etheridge A Piper MG Batte K . Systems biology of interstitial lung diseases: integration of mRNA and microRNA expression changes. BMC Med Genomics. (2011) 4:8. doi: 10.1186/1755-8794-4-8. PMID: 21241464 PMC3035594

[107] Wang B Herman-Edelstein M Koh P Burns W Jandeleit-Dahm K Watson A . E-Cadherin expression is regulated by miR-192/215 by a mechanism that is independent of the profibrotic effects of transforming growth factor-β. Diabetes. (2010) 59:1794–802. doi: 10.2337/db09-1736. PMID: 20393144 PMC2889781

[108] Pogribny IP Starlard-Davenport A Tryndyak VP Han T Ross SA Rusyn I . Difference in expression of hepatic microRNAs miR-29c, miR-34a, miR-155, and miR-200b is associated with strain-specific susceptibility to dietary nonalcoholic steatohepatitis in mice. Lab Invest. (2010) 90:1437–46. doi: 10.1038/labinvest.2010.113. PMID: 20548288 PMC4281935

[109] Dai W Zhao J Tang N Zeng X Wu K Ye C . Micro RNA ‐155 attenuates activation of hepatic stellate cell by simultaneously preventing EMT process and ERK 1 signaling pathway. Liver Int. (2015) 35:1234–43. doi: 10.1111/liv.12660. PMID: 25142507

[110] Lino Cardenas CL Henaoui IS Courcot E Roderburg C Cauffiez C Aubert S . miR-199a-5p is upregulated during fibrogenic response to tissue injury and mediates TGFbeta-induced lung fibroblast activation by targeting Caveolin-1. PloS Genet. (2013) 9:e1003291. doi: 10.1371/journal.pgen.1003291. PMID: 23459460 PMC3573122

[111] Qian Q Ma Q Wang B Qian Q Zhao C Feng F . Downregulated miR-129-5p expression inhibits rat pulmonary fibrosis by upregulating STAT1 gene expression in macrophages. Int Immunopharmacol. (2022) 109:108880. doi: 10.1016/j.intimp.2022.108880. PMID: 35689956

[112] Roderburg C Luedde M Vargas Cardenas D Vucur M Mollnow T Zimmermann HW . miR-133a mediates TGF-β-dependent derepression of collagen synthesis in hepatic stellate cells during liver fibrosis. J Hepatol. (2013) 58:736–42. doi: 10.1016/j.jhep.2012.11.022. PMID: 23183523

[113] Compston A Coles A . Multiple sclerosis. Lancet. (2008) 372:1502–17. doi: 10.1016/S0140-6736(08)61620-7. PMID: 18970977

[114] Lozinski BM Ghorbani S Yong VW . Biology of neurofibrosis with focus on multiple sclerosis. Front Immunol. (2024) 15:1370107. doi: 10.3389/fimmu.2024.1370107. PMID: 38596673 PMC11002094

[115] Ghorbani S Yong VW . The extracellular matrix as modifier of neuroinflammation and remyelination in multiple sclerosis. Brain. (2021) 144:1958–73. doi: 10.1093/brain/awab059. PMID: 33889940 PMC8370400

[116] Mora P Chapouly C . Astrogliosis in multiple sclerosis and neuro-inflammation: what role for the notch pathway? Front Immunol. (2023) 14:1254586. doi: 10.3389/fimmu.2023.1254586. PMID: 37936690 PMC10627009

[117] Yi H Bai Y Zhu X lin L Zhao L Wu X . IL-17A induces MIP-1α expression in primary astrocytes via Src/MAPK/PI3K/NF-kB pathways: implications for multiple sclerosis. J Neuroimmune Pharmacol. (2014) 9:629–41. doi: 10.1007/s11481-014-9553-1. PMID: 24989845

[118] Grygorowicz T Wełniak-Kamińska M Strużyńska L . Early P2X7R-related astrogliosis in autoimmune encephalomyelitis. Mol Cell Neurosci. (2016) 74:1–9. doi: 10.1016/j.mcn.2016.02.003. PMID: 26921791

[119] Sharp AJ Polak PE Simonini V Lin SX Richardson JC Bongarzone ER . P2x7 deficiency suppresses development of experimental autoimmune encephalomyelitis. J Neuroinflamm. (2008) 5:33. doi: 10.1186/1742-2094-5-33. PMID: 18691411 PMC2518548

[120] Mason JL Suzuki K Chaplin DD Matsushima GK . Interleukin-1β promotes repair of the CNS. J Neurosci. (2001) 21:7046–52. doi: 10.1523/JNEUROSCI.21-18-07046.2001. PMID: 11549714 PMC6762979

[121] Steen EH Wang X Balaji S Butte MJ Bollyky PL Keswani SG . The role of the anti-inflammatory cytokine interleukin-10 in tissue fibrosis. Adv Wound Care (New Rochelle). (2020) 9:184–98. doi: 10.1089/wound.2019.1032. PMID: 32117582 PMC7047112

[122] Illanad GH Rithvik A Rasool M . Fibroblast growth factor 2 in the hotbed of rheumatoid arthritis pathogenesis. Int Immunopharmacol. (2026) 168:115778. doi: 10.1016/j.intimp.2025.115778. PMID: 41213186

[123] Zhang XL Topley N Ito T Phillips A . Interleukin-6 regulation of transforming growth factor (TGF)-β receptor compartmentalization and turnover enhances TGF-β1 signaling. J Biol Chem. (2005) 280:12239–45. doi: 10.1074/jbc.M413284200. PMID: 15661740

[124] Yu Z Liu J Chen L Xie J . Role of interleukin-6 in rheumatoid arthritis-associated interstitial lung disease: focus on the JAK/STAT pathway and macrophage polarization. J Inflammation Res. (2025) 18:10953–67. doi: 10.2147/JIR.S530754. PMID: 40827265 PMC12358124

[125] Diarra D Stolina M Polzer K Zwerina J Ominsky MS Dwyer D . Dickkopf-1 is a master regulator of joint remodeling. Nat Med. (2007) 13:156–63. doi: 10.1038/nm1538. PMID: 17237793

[126] Xiong H Wei L Peng B . IL ‐17 stimulates the production of the inflammatory chemokines IL ‐6 and IL ‐8 in human dental pulp fibroblasts. Int Endod J. (2015) 48:505–11. doi: 10.1111/iej.12339. PMID: 25040247

[127] Lee S-Y Kwok S-K Son H-J Ryu J-G Kim E-K Oh H-J . IL-17-mediated Bcl-2 expression regulates survival of fibroblast-like synoviocytes in rheumatoid arthritis through STAT3 activation. Arthritis Res Ther. (2013) 15:R31. doi: 10.1186/ar4179. PMID: 23421940 PMC3672783

[128] Kouri V-P Olkkonen J Nurmi K Peled N Ainola M Mandelin J . IL-17A and TNF synergistically drive expression of proinflammatory mediators in synovial fibroblasts via IκBζ-dependent induction of ELF3. Rheumatology. (2023) 62:872–85. doi: 10.1093/rheumatology/keac385. PMID: 35792833 PMC9891425

[129] Rithvik A Samarpita S Rasool M . Unleashing the pathological imprinting of cancer in autoimmunity: is ZEB1 the answer? Life Sci. (2023) 332:122115. doi: 10.1016/j.lfs.2023.122115. PMID: 37739160

[130] Wijsenbeek MS Kool M Cottin V . Targeting interleukin-13 in idiopathic pulmonary fibrosis: from promising path to dead end. Eur Respir J. (2018) 52:1802111. doi: 10.1183/13993003.02111-2018. PMID: 30545962

[131] Zhu Z Homer RJ Wang Z Chen Q Geba GP Wang J . Pulmonary expression of interleukin-13 causes inflammation, mucus hypersecretion, subepithelial fibrosis, physiologic abnormalities, and eotaxin production. J Clin Invest. (1999) 103:779–88. doi: 10.1172/JCI5909. PMID: 10079098 PMC408149

[132] Peng Y Zhou M Yang H Qu R Qiu Y Hao J . Regulatory mechanism of M1/M2 macrophage polarization in the development of autoimmune diseases. Mediators Inflammation. (2023) 2023:1–20. doi: 10.1155/2023/8821610. PMID: 37332618 PMC10270764

[133] Yu Z Liu J Chen L Xie J . Role of interleukin-6 in rheumatoid arthritis-associated interstitial lung disease: focus on the JAK/STAT pathway and macrophage polarization. J Inflammation Res. (2025) 18:10953–67. doi: 10.2147/JIR.S530754. PMID: 40827265 PMC12358124

[134] Diesler R Cottin V . Pulmonary fibrosis associated with rheumatoid arthritis: from pathophysiology to treatment strategies. Expert Rev Respir Med. (2022) 16:541–53. doi: 10.1080/17476348.2022.2089116. PMID: 35695895

[135] Liu J Xu L Guan X Zhang J . Experimental study of the effects of pirfenidone and nintedanib on joint inflammation and pulmonary fibrosis in a rheumatoid arthritis-associated interstitial lung disease mouse model. J Thorac Dis. (2024) 16:7458–76. doi: 10.21037/jtd-24-882. PMID: 39678895 PMC11635228

[136] Liu H Yang Y Zhang J Li X . Baricitinib improves pulmonary fibrosis in mice with rheumatoid arthritis-associated interstitial lung disease by inhibiting the Jak2/Stat3 signaling pathway. Adv Rheumatol. (2023) 63:45. doi: 10.1186/s42358-023-00325-z. PMID: 37641106

[137] Leavy OC Kawano-Dourado L Stewart ID Quint JK Solomon JJ Borie R . Rheumatoid arthritis and idiopathic pulmonary fibrosis: a bidirectional Mendelian randomization study. Thorax. (2024) 79:538–44. doi: 10.1136/thorax-2023-220856. PMID: 38649271 PMC11137470

[138] Yang X Liu Z Zhou J Guo J Han T Liu Y . SPP1 promotes the polarization of M2 macrophages through the Jak2/Stat3 signaling pathway and accelerates the progression of idiopathic pulmonary fibrosis. Int J Mol Med. (2024) 54:89. doi: 10.3892/ijmm.2024.5413. PMID: 39129313 PMC11335352

[139] Zhang S Zhu P Yuan J Cheng K Xu Q Chen W . Non-alcoholic fatty liver disease combined with rheumatoid arthritis exacerbates liver fibrosis by stimulating co-localization of PTRF and TLR4 in rats. Front Pharmacol. (2023) 14:1149665. doi: 10.3389/fphar.2023.1149665. PMID: 37346294 PMC10279862

[140] Krizhanovsky V Yon M Dickins RA Hearn S Simon J Miething C . Senescence of activated stellate cells limits liver fibrosis. Cell. (2008) 134:657–67. doi: 10.1016/j.cell.2008.06.049. PMID: 18724938 PMC3073300

[141] Negrini S Emmi G Greco M Borro M Sardanelli F Murdaca G . Sjögren’s syndrome: a systemic autoimmune disease. Clin Exp Med. (2022) 22:9–25. doi: 10.1007/s10238-021-00728-6. PMID: 34100160 PMC8863725

[142] Lavoie TN Stewart CM Berg KM Li Y Nguyen CQ . Expression of interleukin‐22 in Sjögren’s syndrome: significant correlation with disease parameters. Scand J Immunol. (2011) 74:377–82. doi: 10.1111/j.1365-3083.2011.02583.x. PMID: 21645026 PMC3250060

[143] Sisto M Lorusso L Tamma R Ingravallo G Ribatti D Lisi S . Interleukin-17 and -22 synergy linking inflammation and EMT-dependent fibrosis in Sjögren’s syndrome. Clin Exp Immunol. (2019) 198:261–72. doi: 10.1111/cei.13337. PMID: 31165469 PMC6797899

[144] Xiao F Du W Zhu X Tang Y Liu L Huang E . IL‐17 drives salivary gland dysfunction via inhibiting TRPC1‐mediated calcium movement in Sjögren’s syndrome. Clin Transl Immunol. (2021) 10. doi: 10.1002/cti2.1277. PMID: 33968407 PMC8082715

[145] Zhou J Onodera S Hu Y Yu Q . Interleukin-22 exerts detrimental effects on salivary gland integrity and function. Int J Mol Sci. (2022) 23:12997. doi: 10.3390/ijms232112997. PMID: 36361787 PMC9655190

[146] Lavoie TN Carcamo WC Wanchoo A Sharma A Gulec A Berg KM . IL-22 regulation of functional gene expression in salivary gland cells. Genom Data. (2016) 7:178–84. doi: 10.1016/j.gdata.2015.11.014. PMID: 26981401 PMC4778602

[147] Mahlios J Zhuang Y . Contribution of IL-13 to early exocrinopathy in Id3-/- mice. Mol Immunol. (2011) 49:227–33. doi: 10.1016/j.molimm.2011.08.012. PMID: 21924496 PMC3205188

[148] Iyer SN Gurujeyalakshmi G Giri SN . Effects of pirfenidone on transforming growth factor-β gene expression at the transcriptional level in bleomycin hamster model of lung fibrosis. J Pharmacol Exp Ther. (1999) 291:367–73. doi: 10.1016/S0022-3565(24)35110-9. PMID: 10490926

[149] Hostettler KE Zhong J Papakonstantinou E Karakiulakis G Tamm M Seidel P . Anti-fibrotic effects of nintedanib in lung fibroblasts derived from patients with idiopathic pulmonary fibrosis. Respir Res. (2014) 15:157. doi: 10.1186/s12931-014-0157-3. PMID: 25496490 PMC4273482

[150] Song M-K Lee J-H Ryoo I Lee S Ku S-K Kwak M-K . Bardoxolone ameliorates TGF-β1-associated renal fibrosis through Nrf2/Smad7 elevation. Free Radic Biol Med. (2019) 138:33–42. doi: 10.1016/j.freeradbiomed.2019.04.033. PMID: 31059771

[151] Luangmonkong T Suriguga S Bigaeva E Boersema M Oosterhuis D de Jong KP . Evaluating the antifibrotic potency of galunisertib in a human ex vivo model of liver fibrosis. Br J Pharmacol. (2017) 174:3107–17. doi: 10.1111/bph.13945. PMID: 28691737 PMC5573419

[152] Krawczyk A Kravčenia B Maślanka T . Mycophenolate mofetil: an update on its mechanism of action and effect on lymphoid tissue. Front Immunol. (2025) 15:1463429. doi: 10.3389/fimmu.2024.1463429. PMID: 39845953 PMC11753233

[153] Pope JE Bellamy N Seibold JR Baron M Ellman M Carette S . A randomized, controlled trial of methotrexate versus placebo in early diffuse scleroderma. Arthritis Rheum. (2001) 44:1351–8. doi: 10.1002/1529-0131(200106)44:6<1351::AID-ART227>3.0.CO;2-I 11407694

[154] Hauser SL Waubant E Arnold DL Vollmer T Antel J Fox RJ . B-cell depletion with rituximab in relapsing–remitting multiple sclerosis. N Engl J Med. (2008) 358:676–88. doi: 10.1056/NEJMoa0706383. PMID: 18272891

[155] Huang R Fu P Ma L . Kidney fibrosis: from mechanisms to therapeutic medicines. Signal Transduct Target Ther. (2023) 8:129. doi: 10.1038/s41392-023-01379-7. PMID: 36932062 PMC10023808

[156] Huang W-C Chuang C-F Huang Y-T Chung I-C Chen M-L Chuang T-Y . Monoclonal enolase-1 blocking antibody ameliorates pulmonary inflammation and fibrosis. Respir Res. (2023) 24:280. doi: 10.1186/s12931-023-02583-3. PMID: 37964270 PMC10647181

[157] Liu J Li H Chen H Xiao X Jin Z Paerhati P . An anti-RAGE chimeric antibody alleviates CCl4-induced liver fibrosis via RAGE/NF-kB pathway in mice. Biomedicine Pharmacotherapy. (2024) 181:117737. doi: 10.1016/j.biopha.2024.117737. PMID: 39657505

[158] Peng X Yang H Tao L Xiao J Zeng Y Shen Y . Fluorofenidone alleviates liver fibrosis by inhibiting hepatic stellate cell autophagy via the TGF-β1/Smad pathway: implications for liver cancer. PeerJ. (2023) 11:e16060. doi: 10.7717/peerj.16060. PMID: 37790613 PMC10542821

[159] Invernizzi P Carbone M Jones D Levy C Little N Wiesel P . Setanaxib, a first‐in‐class selective NADPH oxidase 1/4 inhibitor for primary biliary cholangitis: a randomized, placebo‐controlled, phase 2 trial. Liver Int. (2023) 43:1507–22. doi: 10.1111/liv.15596. PMID: 37183520

[160] Chalasani N Abdelmalek MF Garcia-Tsao G Vuppalanchi R Alkhouri N Rinella M . Effects of Belapectin, an inhibitor of Galectin-3, in patients with nonalcoholic steatohepatitis with cirrhosis and portal hypertension. Gastroenterology. (2020) 158:1334–1345.e5. doi: 10.1053/j.gastro.2019.11.296. PMID: 31812510

[161] Kruger AJ Fuchs BC Masia R Holmes JA Salloum S Sojoodi M . Prolonged cenicriviroc therapy reduces hepatic fibrosis despite steatohepatitis in a diet‐induced mouse model of nonalcoholic steatohepatitis. Hepatol Commun. (2018) 2:529–45. doi: 10.1002/hep4.1160. PMID: 29761169 PMC5944590

[162] Fang R Zhou Z Chu R Guan Q He F Ge M . G protein-coupled receptor kinase 2 as a novel therapeutic target for gland fibrosis of Sjögren’s syndrome. Acta Pharmacol Sin. (2024) 45:2611–24. doi: 10.1038/s41401-024-01350-4. PMID: 39054339 PMC11579508

[163] Sun T Liu S Yang G Zhu R Li Z Yao G . Mesenchymal stem cell transplantation alleviates Sjögren’s syndrome symptoms by modulating Tim-3 expression. Int Immunopharmacol. (2022) 111:109152. doi: 10.1016/j.intimp.2022.109152. PMID: 36007392

[164] Kaieda S Fujimoto K Todoroki K Abe Y Kusukawa J Hoshino T . Mast cells can produce transforming growth factor β1 and promote tissue fibrosis during the development of Sjögren’s syndrome-related sialadenitis. Mod Rheumatol. (2022) 32:761–9. doi: 10.1093/mr/roab051. PMID: 34915577

[165] Peng B Guo X Kang J Pan S Wei L Wang L . Saliva-derived extracellular vesicles: a promising therapeutic approach for salivary gland fibrosis. J Transl Med. (2025) 23:593. doi: 10.1186/s12967-025-06620-1. PMID: 40426212 PMC12107832

[166] Cavalcanti GV de Oliveira FR Bannitz RF de Paula NA Motta ACF Rocha EM . Correction: Endoplasmic reticulum stress in the salivary glands of patients with primary and associated Sjögren’s disease, and non-Sjögren’s sicca syndrome: a comparative analysis and the influence of chloroquine. Adv Rheumatol. (2025) 65:3. doi: 10.1186/s42358-025-00436-9. PMID: 39838498 PMC13488703

[167] Manfredi A Vacchi C DellaCasa G Cerri S Cassone G Di Cecco G . Fibrosing interstitial lung disease in primary Sjogren syndrome. Joint Bone Spine. (2021) 88:105237. doi: 10.1016/j.jbspin.2021.105237. PMID: 34118430

[168] Liang X Li Y Huang D Zhao X Wu Y Liao R . Establishment of a mouse model of Sjögren syndrome–related interstitial lung disease. J Immunol. (2025) 214:2861–70. doi: 10.1093/jimmun/vkaf211. PMID: 40849891

[169] Alfredsson J Wick MJ . Mechanism of fibrosis and stricture formation in Crohn’s disease. Scand J Immunol. (2020) 92:367–373. doi: 10.1111/sji.12990. PMID: 33119150 PMC7757243

[170] Wang Y Huang B Jin T Ocansey DKW Jiang J Mao F . Intestinal fibrosis in inflammatory bowel disease and the prospects of mesenchymal stem cell therapy. Front Immunol. (2022) 13:835005. doi: 10.3389/fimmu.2022.835005. PMID: 35370998 PMC8971815

[171] Lin X Wang Y Liu Z Lin S Tan J He J . Intestinal strictures in Crohn’s disease: a 2021 update. Therap Adv Gastroenterol. (2022) 15:33–42. doi: 10.1177/17562848221104951. PMID: 35757383 PMC9218441

[172] Santacroce G Lenti MV Di Sabatino A . Therapeutic targeting of intestinal fibrosis in Crohn’s disease. Cells. (2022) 11:429. doi: 10.3390/cells11030429. PMID: 35159238 PMC8834168

[173] Speca S . Cellular and molecular mechanisms of intestinal fibrosis. World J Gastroenterol. (2012) 18:3635. doi: 10.3748/wjg.v18.i28.3635. PMID: 22851857 PMC3406417

[174] Liu J Gong W Liu P Li Y Jiang H Wu C . Osteopontin regulation of MerTK+ macrophages promotes Crohn’s disease intestinal fibrosis. iScience. (2024) 27:110226. doi: 10.1016/j.isci.2024.110226. PMID: 39021800 PMC11253513

[175] Biancheri P Pender SL Ammoscato F Giuffrida P Sampietro G Ardizzone S . The role of interleukin 17 in Crohn’s disease-associated intestinal fibrosis. Fibrogenesis Tissue Repair. (2013) 6:13. doi: 10.1186/1755-1536-6-13. PMID: 23834907 PMC3733737

[176] Zhang H-J Zhang Y-N Zhou H Guan L Li Y Sun M-J . IL-17A promotes initiation and development of intestinal fibrosis through EMT. Dig Dis Sci. (2018) 63:2898–909. doi: 10.1007/s10620-018-5234-x. PMID: 30097894

[177] Wang L Wang S Lin J Li J Wang M Yu J . Treg and intestinal myofibroblasts-derived amphiregulin induced by TGF-β mediates intestinal fibrosis in Crohn’s disease. J Transl Med. (2025) 23:452. doi: 10.1186/s12967-025-06413-6. PMID: 40247299 PMC12004752

[178] Wang S Wang L Lin J Wang M Li J Guo Q . Inflammatory monocyte-derived amphiregulin mediates intestinal fibrosis in Crohn’s disease by activating PI3K/AKT. Mucosal Immunol. (2025) 18:989–1000. doi: 10.1016/j.mucimm.2025.05.008. PMID: 40480418

[179] Pinkaew D Martinez-Hackert E Jia W King MD Miao F Enger NR . Fortilin interacts with TGF-β1 and prevents TGF-β receptor activation. Commun Biol. (2022) 5:157. doi: 10.1038/s42003-022-03112-6. PMID: 35197550 PMC8866402

[180] Fuster-Martínez I Calatayud S . The current landscape of antifibrotic therapy across different organs: a systematic approach. Pharmacol Res. (2024) 205:107245. doi: 10.1016/j.phrs.2024.107245. PMID: 38821150

[181] Hasegawa M Hamaguchi Y Yanaba K Bouaziz J-D Uchida J Fujimoto M . B-lymphocyte depletion reduces skin fibrosis and autoimmunity in the tight-skin mouse model for systemic sclerosis. Am J Pathol. (2006) 169:954–66. doi: 10.2353/ajpath.2006.060205. PMID: 16936269 PMC1698806

[182] Lv W Booz GW Fan F Wang Y Roman RJ . Oxidative stress and renal fibrosis: recent insights for the development of novel therapeutic strategies. Front Physiol. (2018) 9:105. doi: 10.3389/fphys.2018.00105. PMID: 29503620 PMC5820314

[183] Petersen AG Korntner SH Bousamaki J Oró D Arraut AM Pors SE . Reproducible lung protective effects of a TGFβR1/ALK5 inhibitor in a bleomycin‐induced and spirometry‐confirmed model of IPF in male mice. Physiol Rep. (2024) 12. doi: 10.14814/phy2.70077. PMID: 39394052 PMC11469938

[184] Lefebvre E Moyle G Reshef R Richman LP Thompson M Hong F . Antifibrotic effects of the dual CCR2/CCR5 antagonist cenicriviroc in animal models of liver and kidney fibrosis. PloS One. (2016) 11:e0158156. doi: 10.1371/journal.pone.0158156. PMID: 27347680 PMC4922569

[185] Chen Y Jiang Q Xing X Xu L Zhao Q Zhang Q . Macrophage derived Galectin‐3 promotes renal fibrosis and diabetic kidney disease by enhancing TGFβ1 signaling. Adv Sci. (2025) 12. doi: 10.1002/advs.202504032. PMID: 40799164 PMC12462920

[186] Chrysanthopoulou A Mitroulis I Apostolidou E Arelaki S Mikroulis D Konstantinidis T . Neutrophil extracellular traps promote differentiation and function of fibroblasts. J Pathol. (2014) 233:294–307. doi: 10.1002/path.4359. PMID: 24740698

[187] He J Cheng X Fang B Shan S Li Q . Mechanical stiffness promotes skin fibrosis via Piezo1-Wnt2/Wnt11-CCL24 positive feedback loop. Cell Death Dis. (2024) 15:84. doi: 10.1038/s41419-024-06466-3. PMID: 38267432 PMC10808102

[188] Hernandez-Gonzalez F Prats N Ramponi V López-Domínguez JA Meyer K Aguilera M . Human senescent fibroblasts trigger progressive lung fibrosis in mice. Aging. (2023) 15:6641–57. doi: 10.18632/aging.204825. PMID: 37393107 PMC10415539

